# Quantification of photoinduced bending of dynamic molecular crystals: from macroscopic strain to kinetic constants and activation energies†
†Electronic supplementary information (ESI) available. CCDC 1536477–1536488. For ESI and crystallographic data in CIF or other electronic format see DOI: 10.1039/c7sc04863g


**DOI:** 10.1039/c7sc04863g

**Published:** 2018-01-22

**Authors:** Stanislav Chizhik, Anatoly Sidelnikov, Boris Zakharov, Panče Naumov, Elena Boldyreva

**Affiliations:** a Institute of Solid State Chemistry and Mechanochemistry , Siberian Branch of Russian Academy of Sciences , ul. Kutateladze, 18 , Novosibirsk 620128 , Russian Federation . Email: csagbox@gmail.com; b Novosibirsk State University , ul. Pirogova, 2 , Novosibirsk 630090 , Russian Federation; c New York University Abu Dhabi , P.O. Box 129188 , Abu Dhabi , United Arab Emirates

## Abstract

Photomechanically reconfigurable elastic single crystals are the key elements for contactless, timely controllable and spatially resolved transduction of light into work from the nanoscale to the macroscale. The deformation in such single-crystal actuators is observed and usually attributed to anisotropy in their structure induced by the external stimulus. Yet, the actual intrinsic and external factors that affect the mechanical response remain poorly understood, and the lack of rigorous models stands as the main impediment towards benchmarking of these materials against each other and with much better developed soft actuators based on polymers, liquid crystals and elastomers. Here, experimental approaches for precise measurement of macroscopic strain in a single crystal bent by means of a solid-state transformation induced by light are developed and used to extract the related temperature-dependent kinetic parameters. The experimental results are compared against an overarching mathematical model based on the combined consideration of light transport, chemical transformation and elastic deformation that does not require fitting of any empirical information. It is demonstrated that for a thermally reversible photoreactive bending crystal, the kinetic constants of the forward (photochemical) reaction and the reverse (thermal) reaction, as well as their temperature dependence, can be extracted with high accuracy. The improved kinematic model of crystal bending takes into account the feedback effect, which is often neglected but becomes increasingly important at the late stages of the photochemical reaction in a single crystal. The results provide the most rigorous and exact mathematical description of photoinduced bending of a single crystal to date.

## Abstract 2

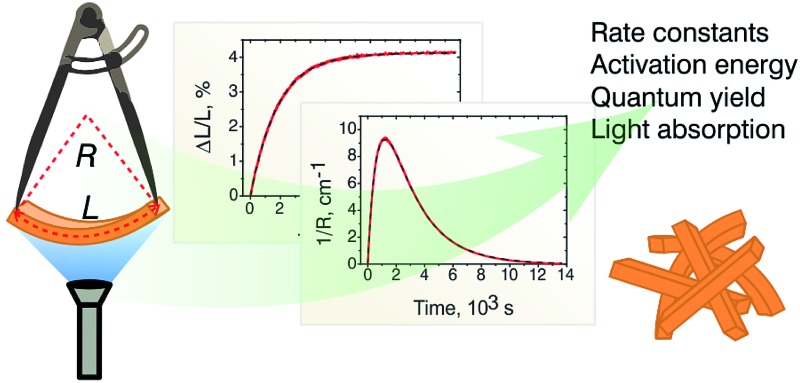
Precise measurement of bending kinematics induced by a photochemical reaction in a single crystal can be used to extract the kinetic parameters of the underlying reaction with high accuracy.

## 1. Introduction

When some molecular crystals undergo physical or chemical transformation, they respond to the stresses and strains generated in their interior by bending, twisting, jumping, creeping, or other types of deformation or motion.^1^–^4^ The research into these mechanical effects and the related solids, recently termed dynamic crystals, is rapidly evolving from the plethora of reports on serendipitous observations to an emerging research direction in materials science. The interest in these materials, however, extends beyond the mere academic curiosity and their visual appeal; there are now attempts to assess and even to gear the properties of dynamic crystals towards specific applications, such as in microactuators controlled by light,^5^ heat or humidity, as macroscopic equivalents of the molecular machines within the realm of supramolecular actuation^6^–^22^ and similar to the polymer actuators.^23^–^35^

The phenomenological approach to chemomechanical effects in crystals, however, has not been paralleled with quantification and modelling of these effects. Deeper understanding of the related phenomena is lacking. Deformation of molecular crystals, for instance, is often reported as mere observation of bending or twisting of certain slender crystals when they are exposed to light or heat, and only minimal explanation on the non-uniformity of the product generated in the lattice of the reactant is provided as the root-cause for the deformation. In an attempt to describe the reasons for deformation of single crystals induced by external stimuli such as light-induced bending or twisting, several reports have attempted to assess the effect on deformation of parameters such as light wavelength/intensity or crystal thickness by using mathematical models.^1^,^36^–^44^ These models rely upon basic engineering principles that describe the deformation of bending beams, and range from very simple and convenient, yet inexact descriptions based on bending of bilayer beams,^39^–^42^ to more involved and comprehensive mathematical models and calculations.^1^,^36^,^38^,^43^,^44^ Yet, these mathematical treatments are often partial; they usually take into consideration only the effect of a particular effector and oftentimes their verification involves empirically fitted parameters to reproduce observed trends in experimental data. A universal model is required that accounts for the effects of all intrinsic (to the crystal) and external (experimental conditions) factors, and would be applicable to a broad range of crystals regardless of the underlying solid-state chemistry. Although being of immediate relevance to the assessment of the actuation performance of single crystals, such an overarching model is currently unavailable and is yet to be established. The assessment of the applicability and exactness of such a holistic mathematical model requires comprehensive and systematic kinematic data collected under controllable and systematically varied conditions on a single reaction system.

It has been already demonstrated that the dependence of the macroscopic strain that develops in the interior of the dynamic crystals on the extent of transformation can be described quantitatively.^45^ Measurements of the temperature-dependent crystal strain can further provide the thermal variation of the kinetic parameters. The kinetics is not only central to understanding of the fundamental mechanisms of the solid-state transformations, but is also the key to achieving an external control over the crystal deformation. This article describes approaches to precise measurement of the macroscopic strain in a single crystal that undergoes a solid-state transformation which can be used to determine the exact kinetic parameters. It is demonstrated—unlike the previously proposed approximate semiempirical approaches that use fitted parameters and do not explicitly account for the thermal variation of the kinetics—that the kinetic constants and their temperature dependence of the forward (photochemical) and the reverse (thermally induced) reactions can be extracted with high accuracy. The possibility to detect and quantitatively characterize the effect of the reaction-induced mechanical effects on the kinetic constants of the reaction (the so-called “feedback effect”), which becomes particularly important at high reaction yields, is also demonstrated. The rigorous mathematical treatment of bending of a single crystal reported here provides the most comprehensive theoretical model to date and scrutinizes the key factors which affect the photomechanical bending of crystals. The determination of the kinematic parameters is essential for comparative benchmarking of the currently available and newly reported materials that are being considered as crystalline microactuators for controlled and efficient conversion of thermal, light and mechanical energy into useful work.

## 2. Results and discussion

### 2.1. Selection of the reaction system and methods

Setting an exact mathematical model for crystal bending and derivation of kinetic parameters from the crystal strain poses a number of requirements to the solid-state transformation and to the crystal:

(a) The reaction must be homogeneous—it should proceed by formation of a series of true solid solutions without phase separation between the reactant and the product;

(b) For maximal mechanical response, the habit of the crystal should be needle-like or an elongated plate (the largest cross-sectional size should be less than 1 : 10 of length) which provides conditions for large and easily measured macroscopic deformation like crystal bending;^1^

(c) For simplicity of the mathematical treatment, the motion should be sole bending, and should be devoid of torsion (twisting). This simple deformation can be accomplished with needle-like crystals, where pure elastic strain (bending, if the crystal is irradiated from only one side, or elongation, if it is irradiated uniformly from both sides) can be induced, provided that specific symmetry requirements for its mechanical properties and the deformation are fulfilled.^46^,^47^ Particularly, it is necessary that neither the transformation nor the lengthwise strain cause shear deformation within the plane of irradiation. This is fulfilled when the longest axis of the needle-like crystal is aligned with one of the principal axes of strain caused by the transformation and the plane of the needle's normal cross-section is also the symmetry plane of mechanical properties of both the reactant and the product;

(d) To avoid preferred accumulation of the product on the crystal surface and to effectively translate the strain into bending, the ratio between the crystal thickness and the light penetration depth should be ≤10 (Note 1 in the ESI†). The extinction coefficient should be low in the spectral range where the reaction is initiated. Values > 10^3^ L mol^–1^ cm^–1^ result in localized transformation in a sub-micrometer-thick layer, and thus very thin crystals (<10 μm) of such samples would be required that are difficult to manipulate in the experiments.

(e) To perform a series of measurements using the same crystal, the reaction should be reversible, either thermally or by inducing a reverse photoreaction with light of different wavelength. By eliminating the data scatter that would be introduced by using different crystals related to defects and other crystal-dependent stochastic factors, this effectively improves the overall data precision over multiple experiments.

It is noteworthy that the above requirements should not be considered as strictly mandatory. The model described here is based on a theoretical framework that is generally applicable to phenomena related to photoinduced mechanical response; it could be additionally adapted or expanded to account for particular features of processes in specific cases. Nevertheless, compliance with the above requirements would ensure conditions to accomplish optimal accuracy.

**Chart 1:**
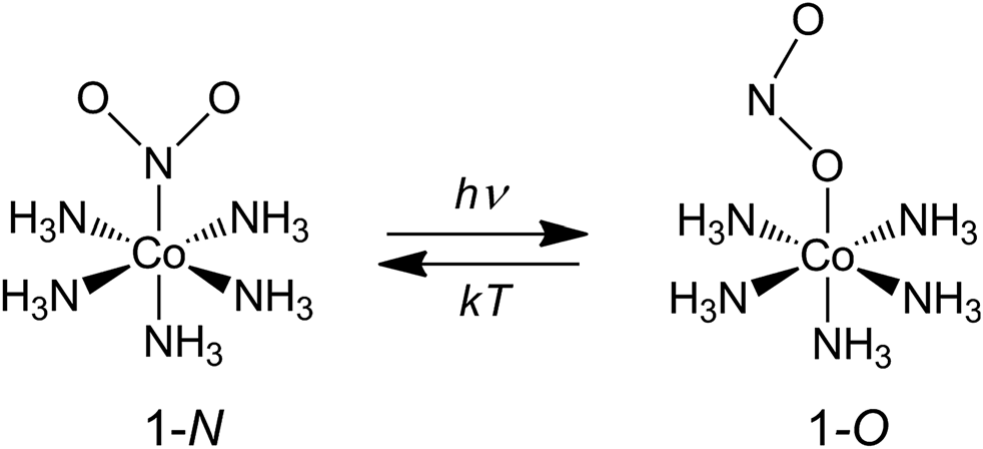
Linkage isomerism in the complex cation [Co(NH_3_)_5_*N*O_2_]^2+^.

Our choice of a solid-state photochemical reaction that conveniently meets all of the above requirements is the reversible linkage isomerization in the needle-shaped crystals of nitropentaamminecobalt(iii) chloride nitrate, [Co(NH_3_)_5_*N*O_2_]Cl(NO_3_) (1-*N*). Upon exposure to blue light, the nitro group in the “nitro form” of this compound switches its coordination atom from nitrogen to oxygen to afford the linkage isomer, the “nitrito form” [Co(NH_3_)_5_*O*NO]Cl(NO_3_) (1-*O*), 1-*N* → 1-*O* (Chart 1). The reverse reaction, 1-*N* ← 1-*O*, can occur by heating^48^,^49^ (ESI Note 2†). Under specific crystallization conditions, crystals of 1-*N* can be grown as needles with the main axis along the [010] (≡*b*) direction, and with (201) and (201) as prominent axial faces. At the local absorption maximum of ∼460 nm, the molar extinction coefficient of a solution of 1-*N* in water is *ε* ≈ 100 L mol^–1^ cm^–1^,^48^,^50^,^51^ which when extrapolated to “concentration” of complex cations in the crystal phase corresponds to characteristic light penetration depth *x*_0_ ≈ 6.5 μm (ESI Note 3†). According to earlier results described in ref. 1, the most prominent mechanical response can be accomplished with crystals of thickness that exceeds *x*_0_ at most several times. This consideration brings a practical convenience with the possibility to use crystals with thickness of several tens of microns that can be easily manipulated by hand. An earlier study^52^,^53^ has established that due to uncompensated internal stress irradiated crystals of 1-*N* thicker than 40–50 μm are readily disintegrated,^54^ and thus ∼40 μm was set as the upper limit for the thickness of the samples in the experiments where uniform irradiation of the crystal from both sides was used to determine the crystal expansion. The crystals used in the experiments with irradiation from one side to determine the degree of crystal bending were thinner than ∼30 μm.

### 2.2. Selection of photoexcitation conditions

**Fig. 1:**
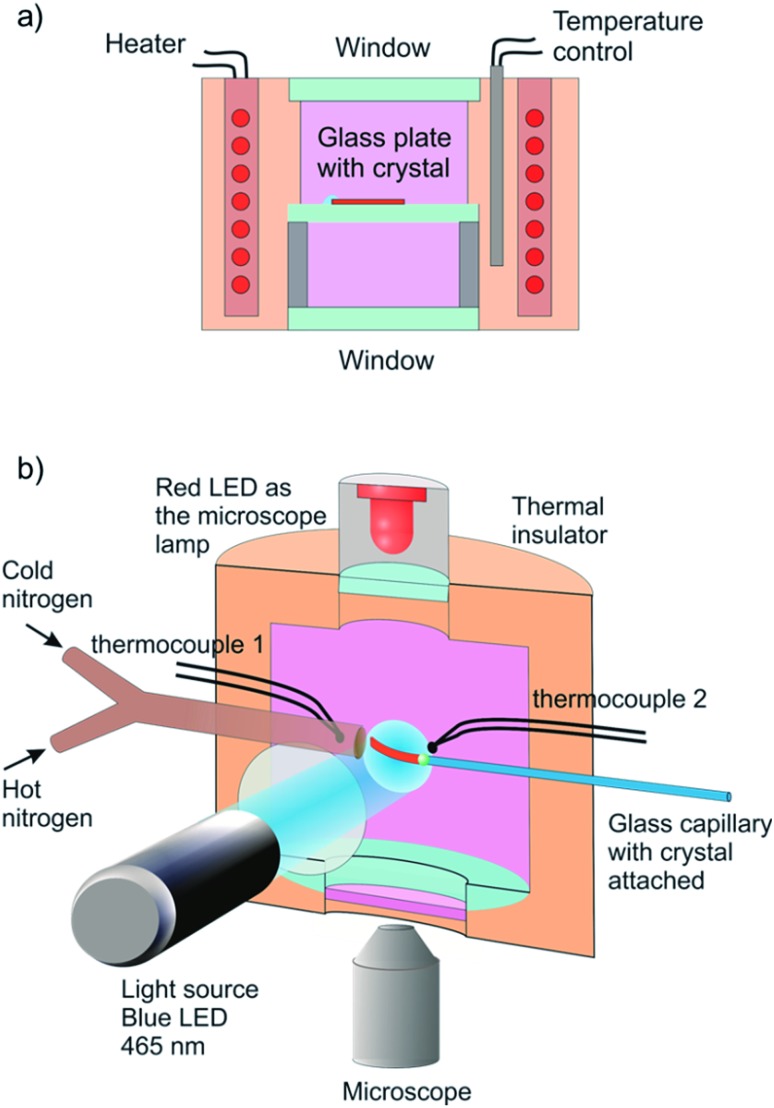
Sketch of the experimental setups used to determine the response of a single crystal to irradiation at controlled temperature. (a) A setup used for measurement of the change in crystal length in the course of the reverse thermal reaction 1-*N* ← 1-*O*. Before the measurement, the crystal was affixed to a glass plate and irradiated with blue (465 nm) LED from both sides while keeping it at –20 °C to accomplish complete transformation. The transformed crystal was positioned inside a thermally controlled chamber and observed with a microscope. (b) A setup for one-side irradiation used to induce and measure bending deformation. A crystal was attached to a capillary in a temperature-controlled chamber and maintained at a constant temperature using streams of hot and cold nitrogen gas. The crystal was irradiated by blue (465 nm) LED to induce the photochemical transformation and observed with a microscope (a red 625 nm LED was used for illumination instead of a standard Xe lamp to prevent undesirable side photoreaction). The crystal size in the figure is exaggerated for clarity.

The reported changes in unit cell parameters upon photoisomerization of 1-*N* at ambient temperature, estimated from X-ray powder diffraction data,^55^,^56^ showed that the structure expands >3% along the crystallographic axis *b* (which coincides with the long axis of the crystal), and contracts significantly in the plane normal to this direction. The strain is sufficient to determine the kinetics at intermediate conversions, and these values have been already used to estimate the relation between the strain and transformation extent.^52^ Two different experimental setups were constructed and used to determine the macroscopic strain that develops upon uniform irradiation of crystals from both sides and from one side only, and are shown in Fig. 1. The two methods result in different distribution of the product of photoisomerization (1-*O*) through the crystal bulk. This was also required to verify if there is any dependence of the reaction constants on the transformation extent, which would result in different constants from the two methods.

#### 2.2.1. Reverse thermal isomerization after prior photoisomerization on uniform irradiation from both sides

A crystal of 1-*N* affixed to a glass plate was uniformly irradiated from both sides at –20 °C for complete transformation to 1-*O* by the photochemical reaction 1-*N* → 1-*O*. The transformed crystal was transferred into a thermal camera maintained at a selected temperature (Fig. 1a). The crystal elongation induced by the photoreaction and subsequent shrinking induced by heating at different temperatures due to the reverse thermal reaction 1-*N* ← 1-*O* was followed by optical microscopy. This method provided the temperature-dependent rate constant (*k*_th_) and activation energy (*E*_th_) of the reverse thermal isomerization. Six different crystals from the same crystallization batch, with lengths ranging between 446 and 1700 μm and thickness between 20 and 30 μm were studied in one or two irradiation-heating cycles. These experiments were performed to assess the reproducibility of the results under conditions of identical temperature for the same crystal, identical temperature for different crystals, different temperatures for the same crystal and different temperatures for different crystals (for the actual combinations between the recorded crystal samples and temperatures, see ESI Table S1†).

#### 2.2.2. Phototransformation and reverse thermal isomerization on uniform irradiation from one side

The crystal was irradiated from one side (one of the long crystal faces was exposed uniformly) to induce bending, and the dynamics of bending during continuous irradiation at various temperatures was determined to calculate the temperature-dependent rate constants *k*_ph_ of the forward reaction, 1-*N* → 1-*O*. Alternatively, to obtain the constants of the reverse thermal isomerization *k*_th_ and the corresponding activation energies, the kinetics of straightening of the crystal due to reverse thermal isomerization was monitored from a photochemically bent crystal. With the goals of the current study requiring the highest precision that could be attained, the same single crystal was used to measure the macroscopic strain (bending) in multiple irradiation-heating cycles in the experiments using the one-side irradiation protocol. We report here only the results obtained for a selected crystal; for a comparison, the data obtained for another crystal are also provided in the ESI (Fig. S5 and S6†).

Single crystals from the same crystallization batch were also used to determine the crystal structure by using single crystal X-ray diffraction before, during and after irradiation—conditions that correspond to pure 1-*N*, solid solutions with varying ratio of the two isomers, and pure 1-*O*, respectively. The variation in unit cell parameters of pure 1-*N* and 1-*O* within the temperature range 175–300 K was also studied. Blue LED (*λ* = 465 nm) was used for excitation (further details are available in the Experimental section and in the ESI†).

### 2.3. Variation of crystallographic parameters with composition and temperature

**Fig. 2:**
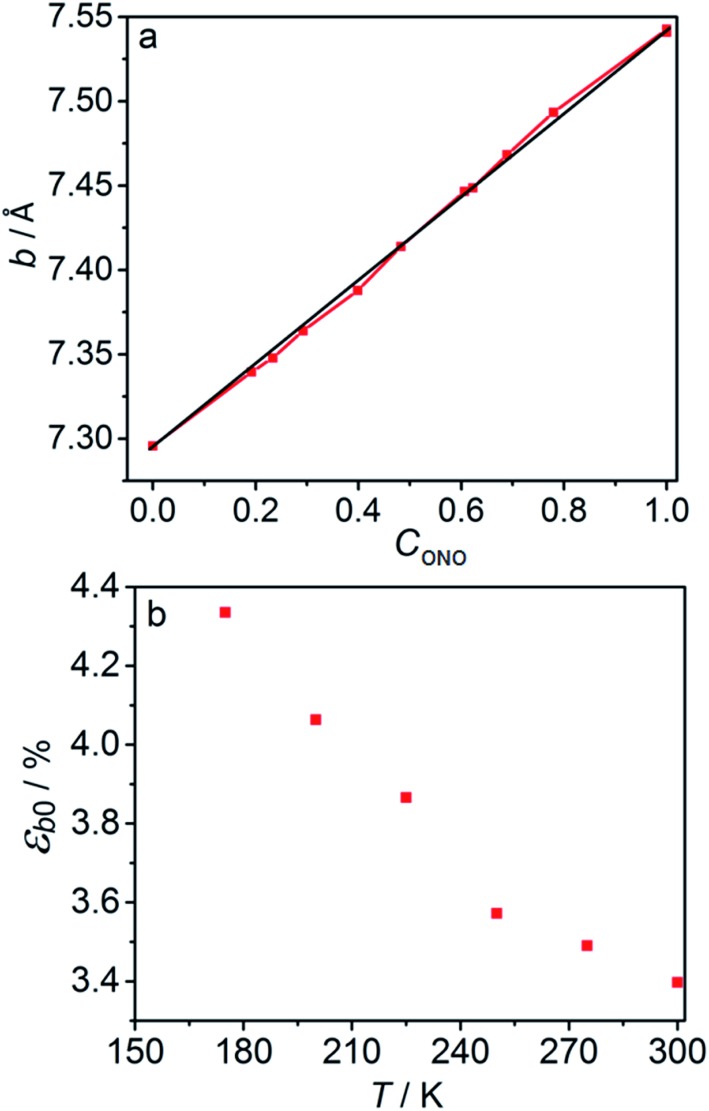
Relation between the long axis *b* and its strain and the reaction extent and temperature. (a) Dependence of the axis *b* on the transformation extent, *C*_ONO_. (b) Temperature dependence of the strain along axis *b*, *ε*_*b*0_, after 1-*N* has been completely converted to 1-*O*.

The macroscopic strain of a needle-shaped crystal, observed as bending or elongation, relates to the definite constituent of lattice deformation caused by the photoinduced transformation, namely the one corresponding to the normal strain developing along the longest crystal axis. In the crystal of 1-*N*, this axis coincides with the crystallographic parameter *b*. Fig. 2a shows the variation of *b* with the concentration of the 1-*O* isomer (*C*_ONO_) determined by refinement of the crystal structure (for details, see Tables S2 and S3 in the ESI†). The dependence of the longitudinal strain along *b*, *ε*_*b*_, on the transformation extent can be approximated by a linear function:1
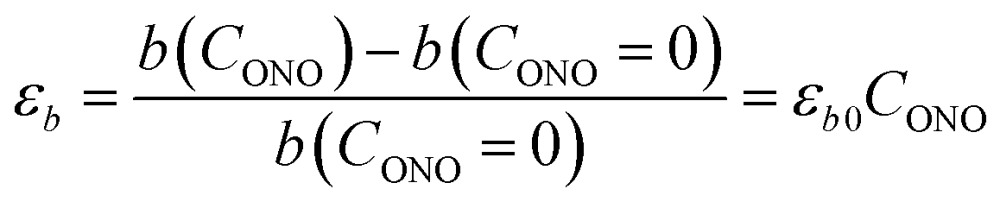
where *ε*_*b*0_ corresponds to the strain along *b* at complete conversion of 1-*N* to 1-*O* (for compositional dependence of the other parameters, see Fig. S2 in the ESI†). In a previous study,^57^ some of us used +3.9% as a rough estimate for *ε*_*b*0_ at ambient temperature based on X-ray powder diffraction data. Generally, this parameter depends on the temperature, since the reactant and the product can have different thermal expansion coefficients. The temperature dependence of *ε*_*b*0_ is shown in Fig. 2b (for temperature dependence of the other parameters, see Fig. S3 in the ESI†). This improved data was used for quantitative analysis of the temperature effects on the crystal strain that evolves during the photoinduced and thermally induced transformations.

### 2.4. Kinetic parameters of the thermal isomerization

#### 2.4.1. Thermal isomerization after irradiation from both sides

**Fig. 3:**
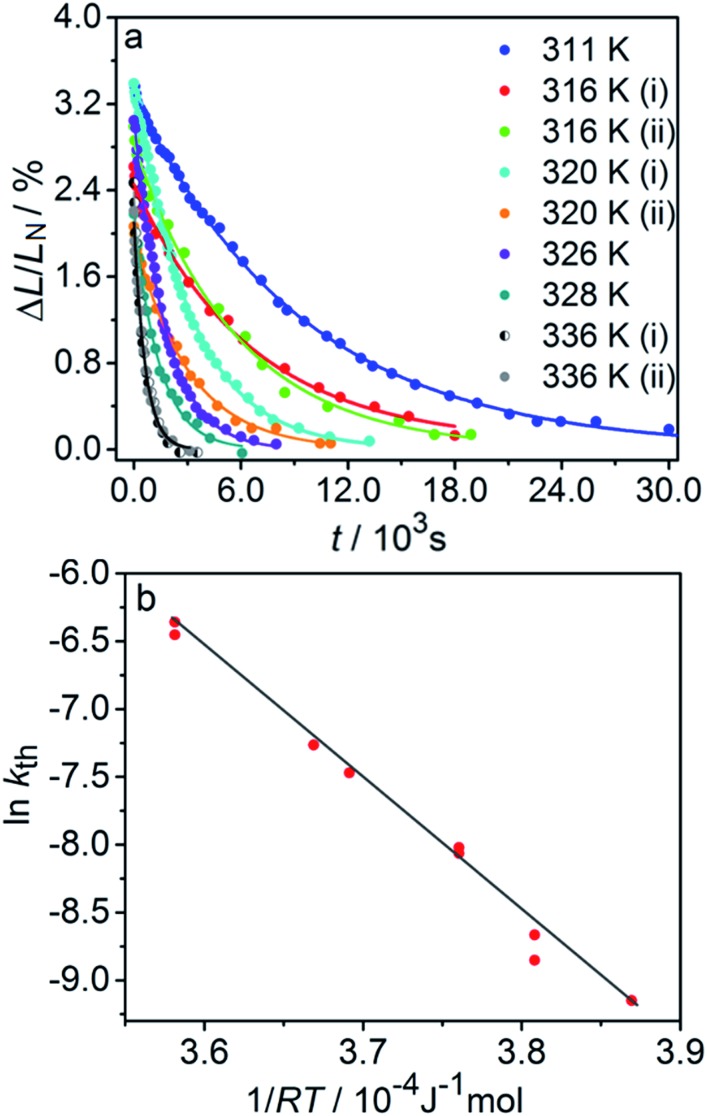
Variation of the crystal length with time and of the rate constant with temperature for the thermal isomerization reaction 1-*N* ← 1-*O*. (a) Change of the relative crystal length Δ*L*/*L*_N_, with time. (b) Temperature dependence of the rate constant (*k*_th_) calculated from the data in panel a to eqn (2).

At complete transformation of 1-*N* to 1-*O*, the elongation of the crystal along its longest axis on uniform irradiation at ambient temperature was always close to 3.4%, in good agreement with the change in the lattice parameter *b* (Section 2.3). On subsequent heating, as the thermal reverse reaction 1-*N* ← 1-*O* occurs, the change Δ*L* = *L* – *L*_N_ in the crystal length *L*, relative to the crystal length *L*_N_ after complete thermal isomerization of 1-*O* to 1-*N* (Fig. 3a) follows the exponential function of time2
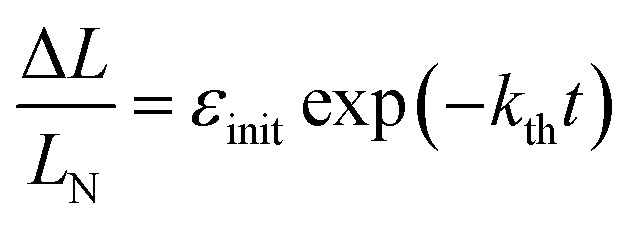
where *ε*_init_ is the initial elongation of the crystal caused by the early uniform photoisomerization to 1-*O*. The crystal strain Δ*L*/*L*_N_ is linearly related to *C*_ONO_ (see Section 2.3). Therefore, the time-dependence of Δ*L*/*L*_N_ given by eqn (2) means that the thermal isomerization 1-*N* ← 1-*O* is a homogeneous monomolecular reaction described by the first-order kinetic law:3
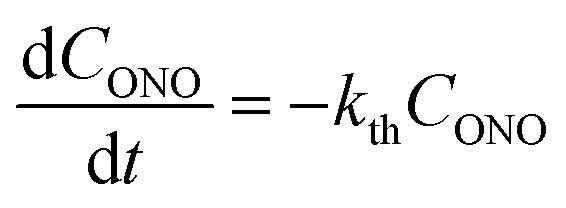



Application of the Arrhenius equation to the temperature dependence of the rate constant *k*_th_ (Fig. 3b) gives activation energy *E*_th_ = 96 ± 2 kJ mol^–1^ and logarithm of the pre-exponential factor ln(*k*0th) = 31.8 ± 0.8 (*k*0th = 7 × 10^13^ s^–1^). These values are consistent with the values Δ*H*_a_ = 81–110 kJ mol^–1^ and *k*0th = 10^10^–10^16^ s^–1^ obtained by using infrared spectroscopy,^58^–^61^ UV-VIS spectroscopy^48^,^62^–^65^ and differential scanning calorimetry^66^ for similar complexes [Co(NH_3_)_5_*O*NO]XY, where X and Y are anions (F^–^, Cl^–^, Br^–^, I^–^, NO_3_^–^) (ESI Note 4†).

#### 2.4.2. Thermal isomerization after irradiation from one side

The kinetics of the thermal isomerization 1-*N* ← 1-*O* was also monitored after the 1-*O* isomer was partially populated by exposing a crystal of 1-*N* to light from one side, using the setup shown in Fig. 1b. Under such conditions, the forward photoreaction 1-*N* → 1-*O* results in bending of the crystal.^53^ The isomerization that corresponds to maximal deflection of the crystal is incomplete, and the concentration of the product (1-*O*) decreases from the exposed surface toward the interior of the crystal due to finite light absorption. Since the relation between the lattice parameter *b* and the transformation extent is linear (see Section 2.3), according to the model described in ref. 1 the curvature of such crystal with non-uniform distribution of the product across its thickness is described as4
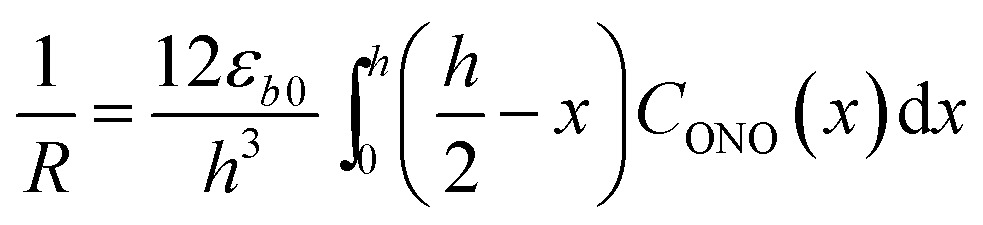
where *R* is the curvature radius, *h* is the crystal thickness, and *C*_ONO_(*x*) is the distribution of the concentration of the product (1-*O*) in the reactant (1-*N*) normal to the irradiated surface and across the depth through the crystal, *x*.

Provided that by subsequent heating of the irradiated crystal in dark the concentration of 1-*O* decreases with time as described with eqn (3), the corresponding change in crystal curvature follows the rate law5
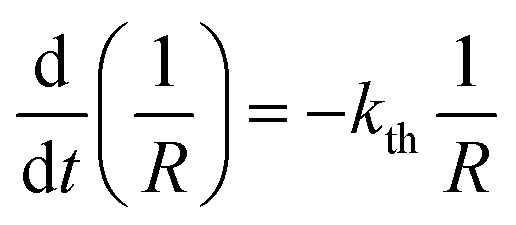



**Fig. 4:**
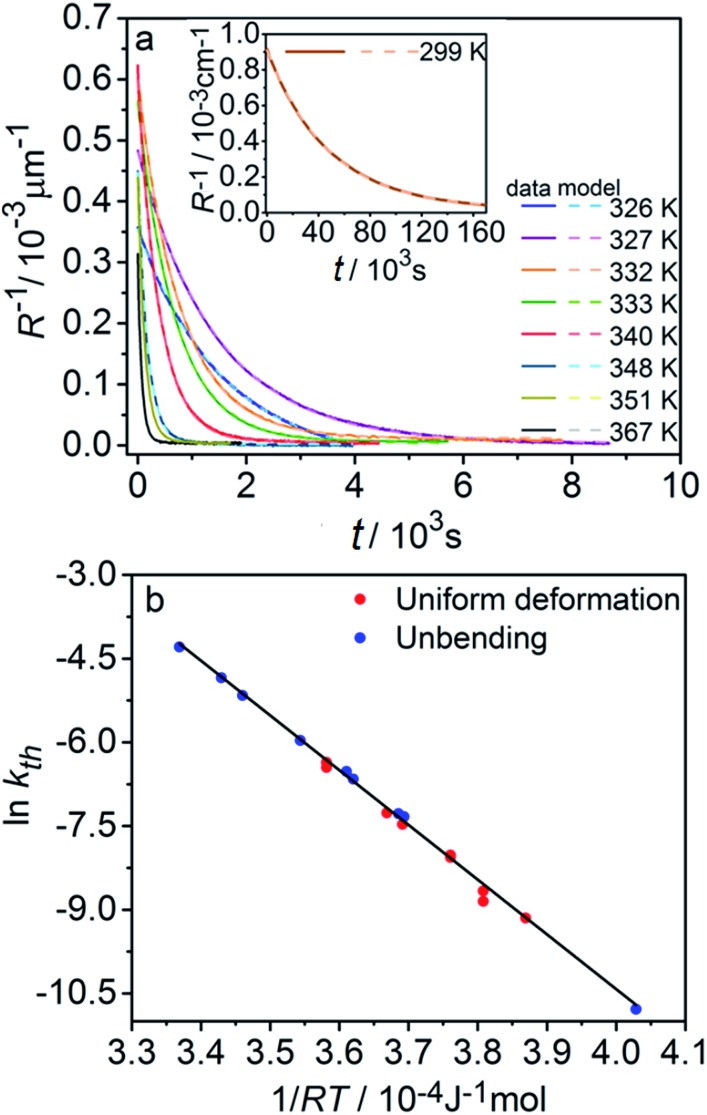
Temperature-dependent dynamics of straightening due to thermal isomerization of a crystal that was bent by irradiation from one side. (a) Experimental data (solid lines) on the change of curvature (1/*R*) over time. The plots are practically indistinguishable from the fits using monoexponential decay functions (broken lines). (b) Dependence of ln(*k*_th_) recorded from uniformly transformed shrinking straight crystal and from thermally induced straightening of a bent crystal on the temperature.

The kinetics of straightening of the crystal is shown in Fig. 4a (for details, see the Experimental section). The time-dependence of the curvature was fitted well by exponential decay functions (ESI Note 5†), and thus the kinetics of the thermal isomerization 1-*N* ← 1-*O* can be safely modelled by a first order rate law. Any dependence of *k*_th_ on the transformation extent could not be detected.

The two different methods to measure the thermal dependence of the kinetics in Fig. 1 provided consistent kinetic constants (ESI Table S4†), activation energy *E*_th_ = 98 ± 1 kJ mol^–1^ and logarithm of the pre-exponential factor ln(*k*0th) = 28.3 ± 0.3 (*k*0th = 3 × 10^12^ s^–1^) (Fig. 4b). These results confirmed that the kinetics of the homogeneous solid-state transformation can be reliably and accurately monitored by measuring the changes in crystal length and/or curvature.

### 2.5. Stationary state of a bent crystal

If a crystal that is being irradiated from one side is simultaneously heated, an equilibrium between the forward and the reverse reaction is eventually established. The bent shape of the crystal can be retained in this stationary state as long as the stationary conditions are maintained.^1^ Although the curvature in the stationary state is less informative relative to the bending kinetics, its dependence on temperature can be used to estimate: (1) the photoisomerization rate at a given temperature, (2) the temperature dependence of the rate of the reverse thermal transformation (*k*_th_), and (3) the characteristic light penetration depth, *x*_0_ = *μ*^–1^, where *μ* is the absorption coefficient in the Beer–Lambert law, *I* = *I*_0_ exp(–*μx*).

The equilibrium established between the photoinduced and thermal transformations results in a stationary spatial gradient of the 1-*O* isomer in the crystal bulk that decreases from the irradiated surface in the direction normal to the surface. For the limiting case of a thin crystal (*μh* ≪ 1), the model described in ref. 1 predicts the stationary curvature *R*^–1^ as6
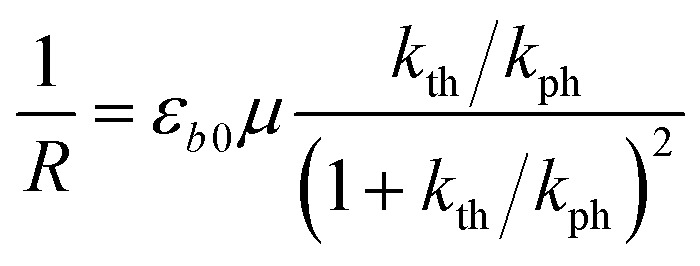



The photoreaction rate constant at the irradiated surface is *k*_ph_ = *I*_0_*αμv*_0_, where *I*_0_ is the normal incident photon flux density, *α* is the quantum yield of the reaction, and *v*_0_ is the reciprocal concentration of the photoreacting species (volume per formula unit). This equation predicts a symmetric bell-like shape of the dependence of the curvature on ln(*k*_th_/*k*_ph_), with a maximum at *k*_th_ = *k*_ph_ (ESI Note 6†).

**Fig. 5:**
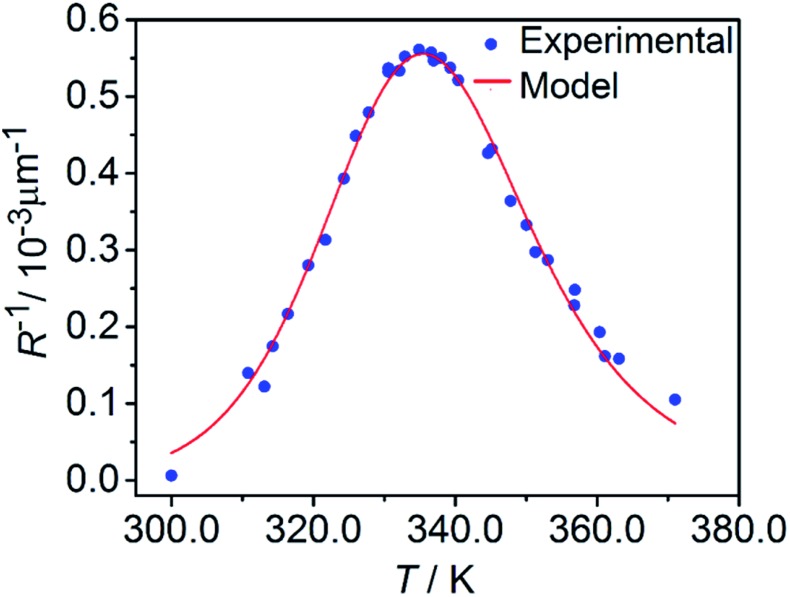
Temperature dependence of the stationary curvature 1/*R* fitted with eqn (6) for Arrhenius-type variation of *k*_th_/*k*_ph_ with temperature according to eqn (7).

The temperature dependence of the stationary curvature of the crystal is shown in Fig. 5 along with a fit based on an Arrhenius-type temperature dependence:7
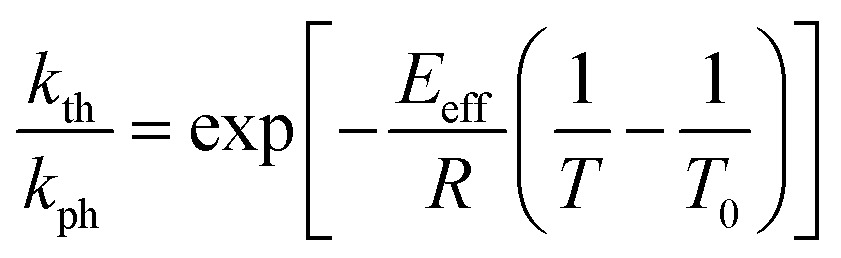



The effective activation energy of the reaction rate ratio, *E*_eff_ = 97 ± 1.5 kJ mol^–1^, is very close to *E*_th_, the activation enthalpy of *k*_th_. This result implies that the photoreaction constant *k*_ph_ is less affected by the temperature and the transformation extent relative to the thermal rate constant *k*_th_, in line with the conclusions in ref. 57. For the light intensity used in this study, the rates of the two reactions (forward and reverse) become identical at *T*_0_ = 335.4 ± 0.2 K. The effective photoreaction rate constant *k*_ph_ depends on the light intensity (proportional to *I*_0_, as noted above), and due to the dependence of the quantum yield it also depends, to a lower extent, on the temperature. Using the determined temperature dependence of *k*_th_ and considering that in the stationary state *k*_th_ = *k*_ph_, the value of *k*_ph_ at this temperature and the given intensity of the radiation source is estimated to ∼1.7 × 10^–3^ s^–1^. Under the given conditions of temperature and irradiation intensity, this value corresponds to half-life of the nitro isomer 1-*N* close to the surface of the crystal of about 400 s.

### 2.6. Estimation of the average absorption coefficient

The absorption coefficient *μ* can be estimated from the experimental data plotted in Fig. 5 by using eqn (6). The maximal stationary curvature given by eqn (6) is *με_b_*_0_/4. From the curve fit in Fig. 5 and using *ε*_*b*0_ = 0.034 (deformation reached on complete transformation at ambient temperature, Section 2.3) the characteristic light penetration depth is estimated to *x*_0_ = *μ*^–1^ ≈ 15 μm, in agreement with the value 6.5 μm estimated from direct measurements of the extinction coefficient in water solution *ε* ≈ 100 L mol^–1^ cm^–1^.^48^,^50^,^51^ Since generally the reactant and the product have different propensities to absorb light, the overall light absorption by the crystal varies with time during the reaction. Thus, this estimated value corresponds to averaged absorption in the stationary state that is reached at *k*_th_ = *k*_ph_. Under this condition, the stationary extent of transformation at the irradiated crystal surface is 0.5 and decreases in the crystal interior due to light absorption. The estimate of the absorption coefficient thus corresponds to average transformation extent of slightly less than 0.5.

### 2.7. Measuring and modelling kinematics of crystal bending under irradiation from one side

#### 2.7.1. Change of transformation extent with time: average gradient and average value

The studies of the kinematics of photoinduced bending of crystals are traditionally limited by an analysis of crystal curvature alone.^39^,^45^ Another independent parameter that characterizes the mechanical response is crystal expansion or contraction along its longest axis but this parameter is usually not considered. The quality of analysis can be enhanced by combining the measurement of the two parameters characterizing the crystal deformation, *i.e.* the curvature and the length of the crystal (for details of the procedure see Section 4.2).

The relative change in crystal length is defined by the average strain *ε*_*b*_ across the crystal thickness:8
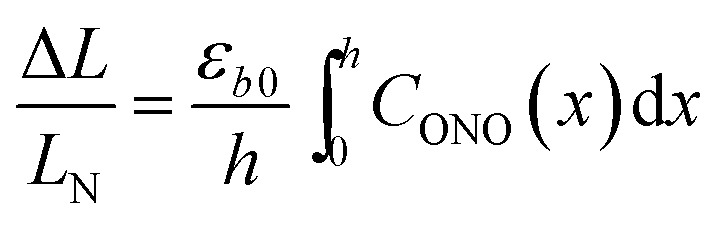



Analysis of the temporal variation of curvature and length using eqn (4) and (8) provides information on two independent characteristics of the distribution of the transformation degree *C*_ONO_(*x*). The crystal elongation in eqn (8) is proportional to the average concentration of 1-*O* across the crystal thickness. The curvature in eqn (4) is proportional to the bending moment caused by non-uniformity of *C*_ONO_(*x*). A linear approximation such as *C*_ONO_(*x*) ≈ *C*_0_ + *x*(d*C*/d*x*)_0_ can be used to demonstrate the qualitative meaning of the integral in eqn (4); the curvature then is 1/*R* ≈ –*ε*_*b*0_(d*C*/d*x*)_0_, that is, it is approximately proportional to the gradient of *C*_ONO_(*x*) over the crystal depth. Thus, the temporal dependence of the crystal elongation and curvature carry information on the average transformation degree and gradient of *C*_ONO_(*x*) as they change over time.

**Fig. 6:**
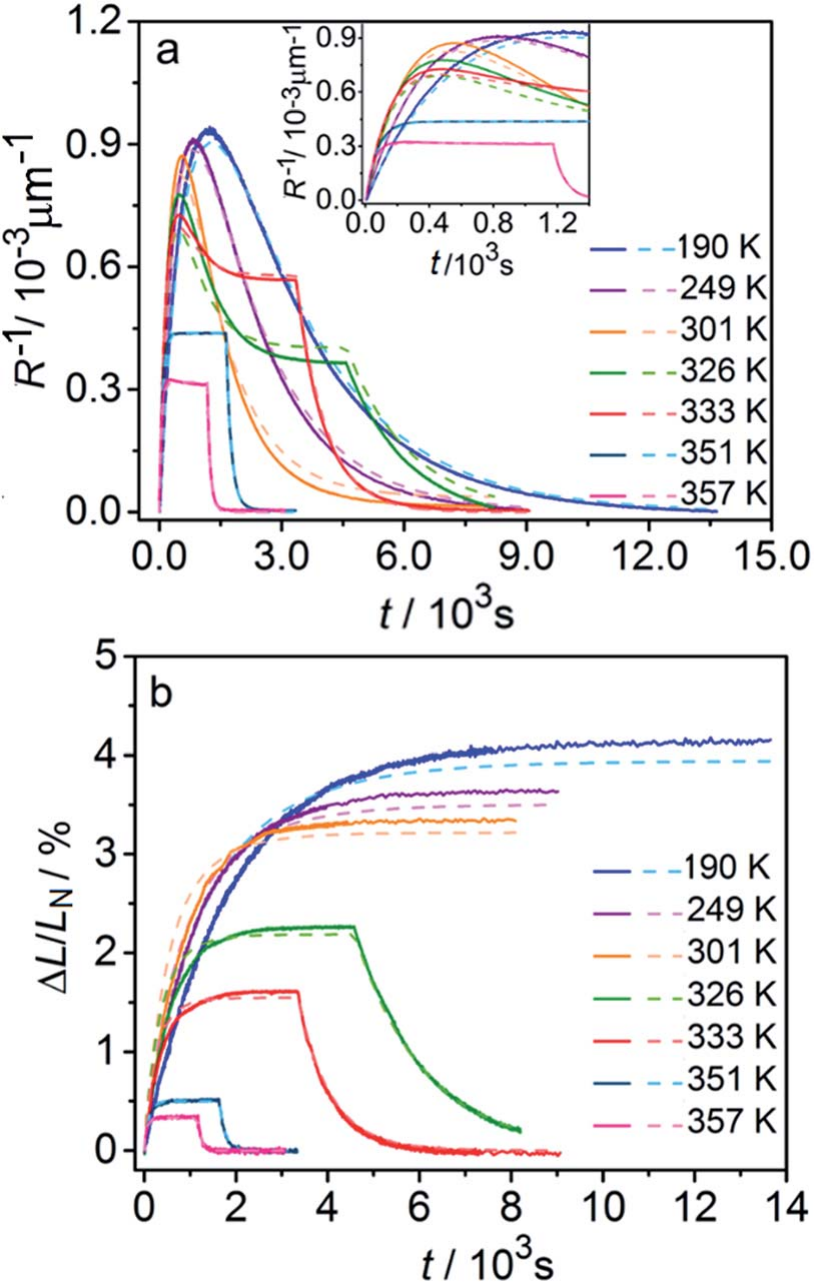
Time- and temperature-dependence of the crystal curvature, *R*^–1^ (a) and the relative elongation, Δ*L*/*L*_N_ (b) under continuous irradiation. In the experiments performed above 326 K, the light was switched off once the stationary state had been reached to record the changes caused by the reverse (thermal) reaction (hence, sharp drops are seen on the respective curves). All experimental data (solid line) and calculations based on the model described by eqn (4), (8) and (11) (broken lines) shown here refer to only one single crystal, in the main text referred to as crystal 1 (similar data for a second crystal, crystal 2, are available as Fig. S5 from the ESI†). The inset in panel a shows a zoomed image of the plot of the curvature at short irradiation times.

Fig. 6 compares the changes in crystal curvature and length over time at different temperatures during continuous irradiation. As it can be inferred from there, while the crystal expands until it reaches a plateau (Fig. 6b), the curvature reaches a maximum and decreases afterwards (Fig. 6a). This change reflects the trend in transformation—while the average transformation extent grows monotonically on irradiation, the absolute value of its gradient initially increases and subsequently decreases. The final curvature corresponds to the stationary state described above. As shown in Fig. 6a, while below ambient temperature the crystal straightens almost completely on prolonged irradiation, at higher temperatures it remains bent. The peak in curvature decreases with temperature and vanishes completely above 350 K. The bending kinetics at 333 K corresponds to conditions close to the maximum stationary bending (Fig. 5).

The behavior of the crystals at and below ambient temperature is in agreement with the results from the diffraction experiments which confirmed that at these temperatures, 1-*N* is completely transformed to 1-*O*. At *T* < 300 K, the reaction can be considered pure photoisomerization, and the reverse (thermal) reaction can be neglected. The maximum elongation of the crystal increases as the temperature decreases (Fig. 6b), in accord with the results in Fig. 2b. The initial rates of the change of crystal length and curvature decrease with decreasing temperature (Fig. 6a), indicating that the rate of photoisomerization decreases. This result is in a qualitative agreement with the dependence of the quantum yield on temperature.^57^

The preceding analysis is rather qualitative and does not provide information on the actual dependence of the absorption coefficient *μ* on temperature or the concentration *C*_ONO_. The rate of change of crystal length and curvature (characterized by the constant *k*_ph_ ∼ *μα*), and the maximum curvature *R*–1max ∼ *με*_*b*0_ are proportional to *μ*. However, they exhibit opposite dependence on the temperature, in agreement with the temperature variation of *α* and *ε*_*b*0_. This result warrants quantitative analysis of the dynamics of mechanical response with transformation.

#### 2.7.2. A general kinetic reaction model

The kinetic model proposed in ref. 1 is based on the equation9
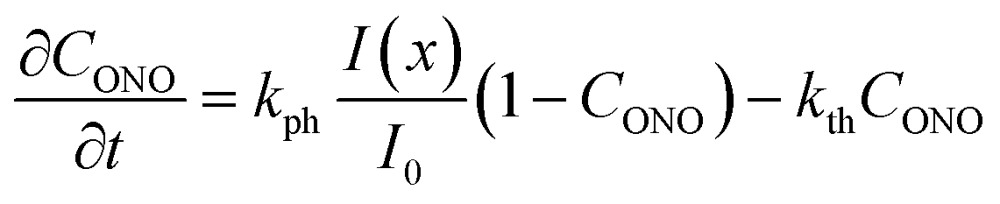
that describes the simultaneous occurrence of two opposite monomolecular reactions. The rate of transformation at distance *x*′ from the surface of the crystal is proportional to the local intensity of the penetrating radiation:10
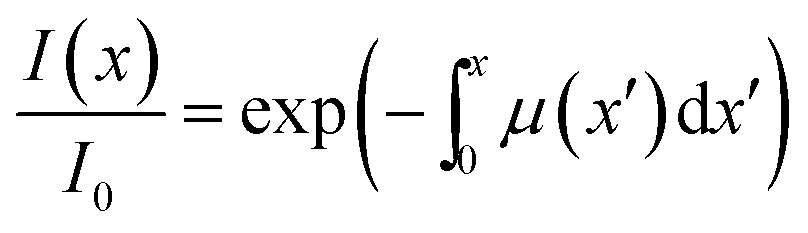



Generally, the absorption coefficient *μ* can vary with the depth because the absorption depends on the extent of transformation (the reactant and product generally have different absorption coefficients). The kinetics of transformation is fully accounted for by this model through three parameters (*k*_ph_, *k*_th_ and *μ*) and by their dependence on the transformation extent. The strain *ε*_*b*0_ relates the kinetics of transformation to the observed mechanical response (eqn (4) and (8)). Thus, only four parameters are necessary for quantitative description of the dynamics of the bending induced by one-side irradiation. As shown above, of these four parameters, *k*_th_ and *ε*_*b*0_, depend only on temperature and can be determined experimentally by using other experimental methods. As the photoreaction constant is defined as *k*_ph_ = *I*_0_*αμv*_0_ and the values of *v*_0_ and *I*_0_ are available independently, the most essential parameters that need to be determined are the quantum yield of the photoreaction *α* and the absorption coefficient *μ*, together with their dependencies on the temperature and on the conversion extent.

The dependence of *μ* on the extent of transformation is mainly determined by the different propensity of the two isomers for light absorption. Other contributions could arise from the different propensity for light absorption of different chromophores (different [Co(NH_3_)_5_*N*O_2_]^2+^ cations in the case of the reaction studied here) in the lattice due to their orientation relative to the direction of the incident light. The quantum yield *α* depends on the lattice strain, as has been previously demonstrated by the effect of external mechanical load^52^ and thermal strain^57^ on the photoisomerization rate. As the progressing transformation also causes strains inside the crystal, it is anticipated that *α* is also affected by the transformation degree.

The inclusion of both thermal and compositional variations of *μ* and *α* in the model adds significantly to the complexity of the mathematical description. In case when *μ* and *α* depend only on temperature and are independent of *C*_ONO_, the differential equation given by eqn (9) can be solved by analytical integration,^1^ which greatly simplifies the subsequent analysis. However, when *μ* and *α* are also affected by the composition, the system of nonlinear equations (eqn (9) and (10)) can only be solved numerically to account for the effect of *C*_ONO_ (this situation is equivalent with the inclusion of the feedback effect, as described above). In order to assess the relevance of these additional contributions, in what follows, a simplified model where *μ* and *α* depend only on temperature is considered first. This simplified model is then followed by a more elaborate analysis that takes into account the variation of the kinetic parameters with the transformation extent.

#### 2.7.3. Quantitative analysis of the kinetics of transformation (*μ* and *α* depend only on temperature)

Assuming that *μ* and *α* depend only on temperature, the solution to eqn (9) is11
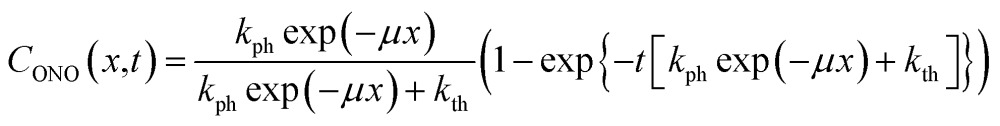



The experimental data on crystal curvature and elongation upon photoreaction at different temperatures fitted with eqn (4), (8) and (11) to obtain temperature dependencies of *ε_b_*_0_, *μ*, and *k*_ph_ are shown in Fig. 6 (the values of *k*_th_ were taken from the results obtained in Section 2.4). As it can be inferred from there, although this simplified treatment provides reasonable quantitative description of the experimental data, there is discrepancy of about 10% in the values of both the curvature and elongation (note the difference between the solid and the dashed lines on both panels in Fig. 6). This discrepancy indicates that the neglecting the dependence of *μ* and *α* on *C*_ONO_ is an oversimplification, and calls for a more sophisticated model that would relate these two parameters with the product concentration. Assuming that the volume per one complex cation is *v*_0_ = 0.26 nm^3^ based on crystallographic data, and approximating the incident photon flux density to *I*_0_ = 1.5 × 10^17^ s^–1^ cm^–2^ (465 nm, photon energy of 2.67 eV), the quantum yield of photoisomerization estimated from the temperature dependence of the fit is *α* = *k*_ph_/*I*_0_*μv*_0_ and ranges between 6% and 16% at temperatures from 190 K to 357 K.

**Fig. 7:**
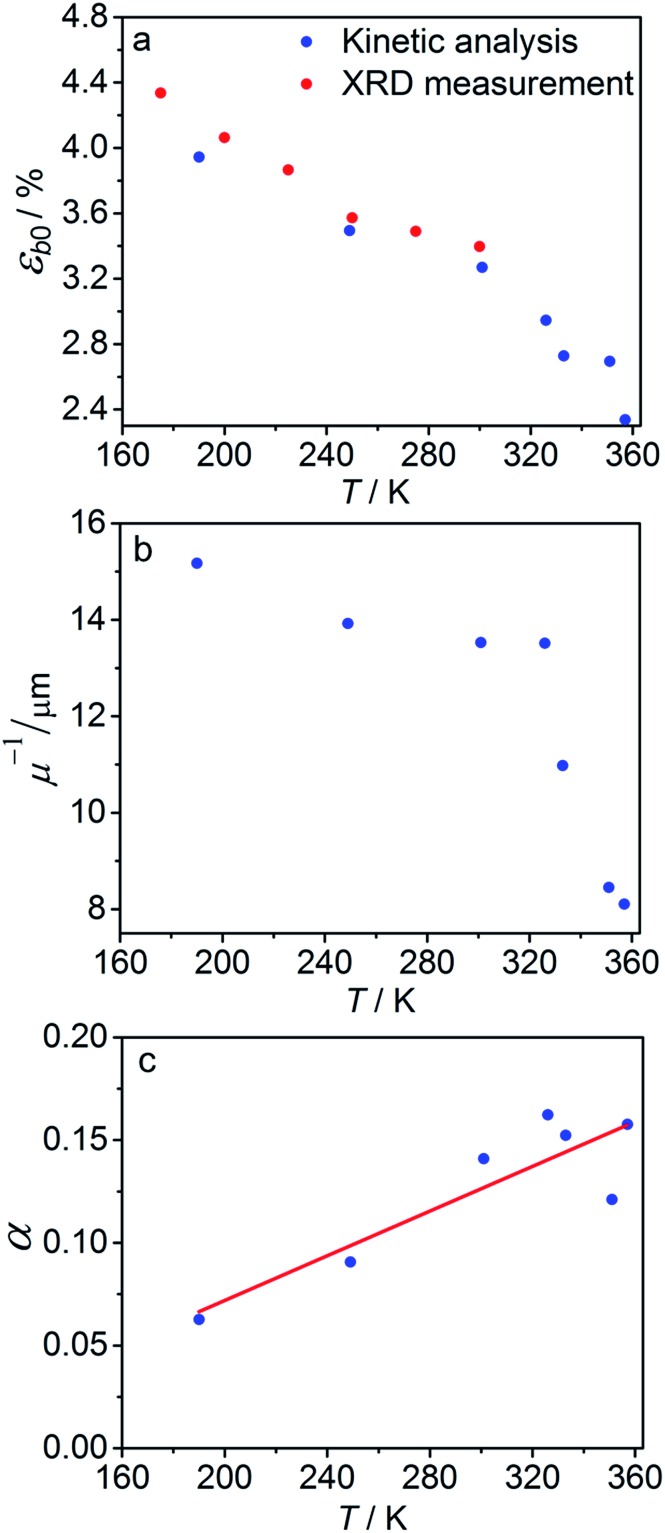
Temperature dependence of the maximum deformation *ε*_*b*0_ (a), characteristic penetration depth (reciprocal of the absorption coefficient *μ*) (b), and quantum yield *α* (c) obtained using the simple kinetic model given by eqn (11) that accounts only for the dependence on the temperature and disregards the variation with the composition.

The temperature dependence of *ε*_*b*0_, *μ*^–1^ and *α* obtained from the fit is shown in Fig. 7. The values of the maximum deformation *ε*_*b*0_ are in agreement with the experimentally determined structure, although the deformation predicted by analysis of the macroscopic strain is systematically underestimated at low temperatures (Fig. 6b), and the trend is different at *T* > 300 K. The temperature trend of the quantum yield corresponds to that obtained in a previous study.^57^ According to ref. 51 and 67 the total quantum yield of photodecomposition and photoisomerization of the complex in water or 50% ethanol in water mixture is 0.15 for excitation around 465 nm. This value is a reasonable estimate for the total quantum yield in the solid state, because both processes ostensibly are initiated from the same excited state, and the decomposition in the solid state is negligible.^68^ Thus, this estimation of the photoisomerization quantum yield from the macroscopic strain is reasonably close to the experimental values obtained by measurements in solution.

The temperature dependence of the quantum yield *α* and the absorption coefficient *μ* (Fig. 7) can now be rationalized. The increase of *α* with temperature can be the consequence of the lattice thermal expansion.^57^ Additional contribution to the high value of *α* at high temperatures could come from the decrease in the overall isomerization extent (ESI Note 7†). The stronger absorption *μ* at higher temperature can also be a consequence of the decreased average reaction extent. As shown in Fig. 7b, the temperature dependence of the penetration depth has two characteristic regimes separated around 330 K. This temperature is close to the point at which the rates of the forward (photochemical) and reverse (thermal) reactions become equal. At lower temperatures, the light is mainly absorbed by the 1-*O* form while at higher temperatures it is mainly absorbed by the initial 1-*N* form. Therefore, the temperature dependence of the absorption coefficient can be a consequence of its variation with composition. This conclusion is confirmed by the lower absorption of the 1-*O* relative to the 1-*N* form around 465 nm.^51^

#### 2.7.4. Improved model for the dynamics of photoinduced bending

With the extensive experimental results at hand, the model proposed several years ago^1^ can now be improved to describe quantitatively the macroscopic deformation of crystals upon photoinduced and thermally induced transformation at variable temperature. The major improvement is the introduction of the dependence of quantum yield *α* and absorption coefficient *μ* on temperature and transformation extent. In the improved model we assume that the quantum yield depends on temperature and transformation that has been already initiated in the crystal:

12 *α* = *α*_0_(*T*)*f*_*α*_(*C*_ONO_, *ε*_r_)

The factors in eqn (12) approximate the environmental effect that the deformation of the crystal structure imposes on the photoreaction.

The first term, the coefficient *α*_0_(*T*), is uniform through the crystal bulk and reflects the dependence of *α* in the initial (non-reacted) crystal on temperature due to thermal expansion (it was shown earlier^57^ that in the linkage isomerization reaction studied here, *α* is linearly related to the change of the unit parameter *a* with temperature). The second term, *f*_*α*_, on the other hand, varies across the crystal from the surface to the bulk. It reflects the dependence of *α* on the local structure deformation caused by the local transformation extent *C*_ONO_(*x*) and by the residual strain *ε*_r_(*x*) of the crystal along its main axis (*b*) related to nonlinearity in *C*_ONO_(*x*). The dependence of *f*_*α*_ on the local transformation degree is qualitatively analogous to the effect of thermal strains described above, but varies across the crystal. The dependence on *ε*_r_ reflects the experimental observation of the effect of external load on the photoisomerization rate.^52^ The residual strain is calculated from the elastostatic solution to the problem of beam bending due to non-uniform distribution of the transformation extent through the crystal bulk:13
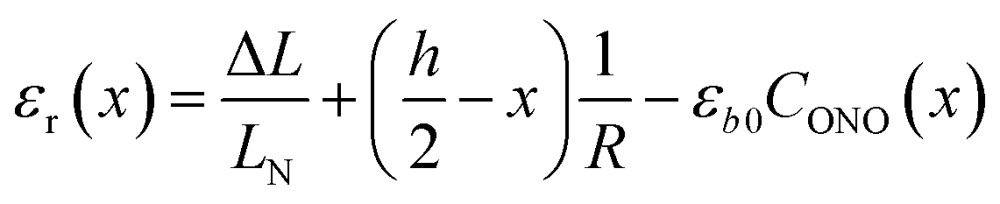



The factor *f*_*α*_ defines the so-called “feedback effect” of the solid state reaction.^69^–^73^ To facilitate the computations, it is convenient to represent *f*_*α*_ as

14 *f*_*α*_ = exp(*q*_*a*_*C*_ONO_ + *q*_*b*_*ε*_r_)

This function approximates the linear dependence of the quantum yield on the strain, provided the arguments of the exponential function are small.^57^ The feedback coefficient *q*_*a*_ can be treated as a fitting parameter of the improved model, while the value of the coefficient *q*_*b*_ is taken to be 40 from the direct measurement in ref. 45. The residual deformation *ε*_r_ does not exceed 0.2*ε*_*b*0_ for crystals that are not thicker than 5*μ*^–1^. Thus, the second term in the exponent for crystals used in the present study is not higher than 0.3.

Probably the simplest way to account for the dependence of the absorption upon the transformation extent is to introduce two absorption coefficients for different isomers, *μ*_0_ for 1-*N* and *μ*_1_ for 1-*O*. Then, the dependence of the local photon flux density on the depth *x* is15
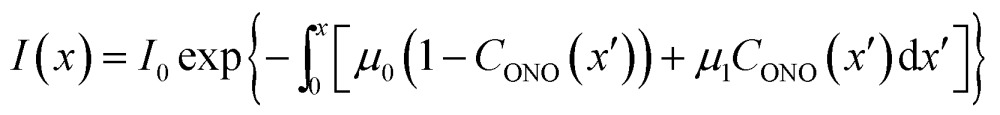
and the kinetic equation is16
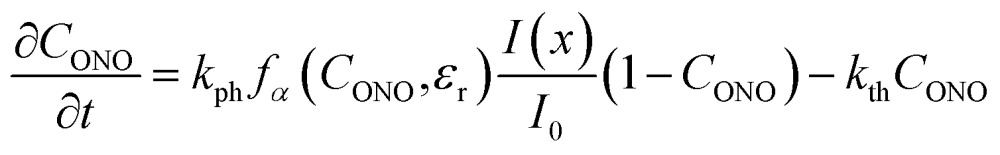
where *k*_ph_ = *I*_0_*α*_0_(*T*)*μ*_0_*v*_0_.

**Fig. 8:**
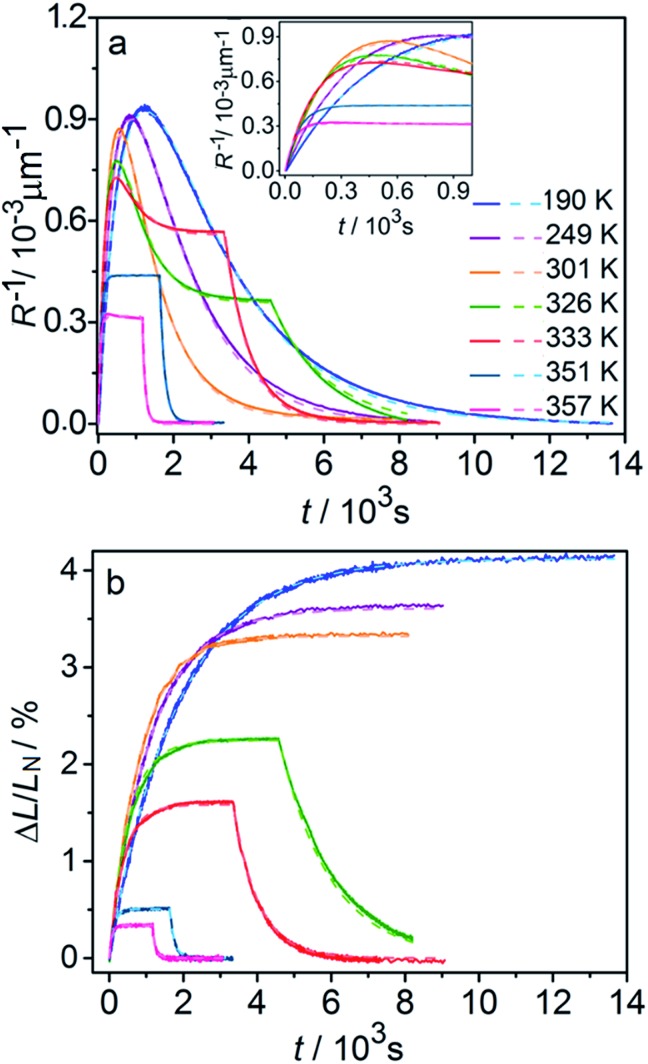
Comparison of the experimentally measured crystal curvature (a) and linear strain (b) with simulations based on the advanced model (eqn (15) and (16)). Note the significant improvement in the fits compared to Fig. 6.

Fig. 8 compares the trends calculated by using the advanced kinetic model (eqn (15) and (16)) and the experimental data. It is apparent that the advanced model provides improved description of the experimental data, and indeed the relative residuals do not exceed 2%. Assuming that the absorption coefficient *μ*_1_ and the feedback coefficient *q*_*a*_ are common fitting parameters for all curves, the fitting procedure results in values for the characteristic penetration depth and feedback coefficient *μ*_1_ = 217 ± 23 cm^–1^ (penetration depth *μ*_1_^–1^ = 46 ± 5 μm) (ESI Note 8†) and *q*_*a*_ = –0.78 ± 0.05, respectively.

The feedback coefficient *q*_*a*_ is related to the effect of lattice strain on the reaction course caused by isomerization. This effect can be compared with the effect of thermal strain on the photoisomerization rate.^57^ The photoisomerization reaction rate and the thermal deformation *ε*_*aT*_(*T*) of the lattice along axis *a* are related as^57^

17 *k*_ph_(*T*) = *k*_ph_(363 K)[1 + *q*_*T*_(*ε*_*aT*_(*T*) – *ε*_*aT*_(363))]

where *q*_*T*_ = 55 ± 5. This dependence can be traced back to the dependence of the quantum yield on the thermal strain, as described in ref. 57. The dependence of *α* on the degree of transformation (eqn (12) and (14)) can be represented in a similar form18
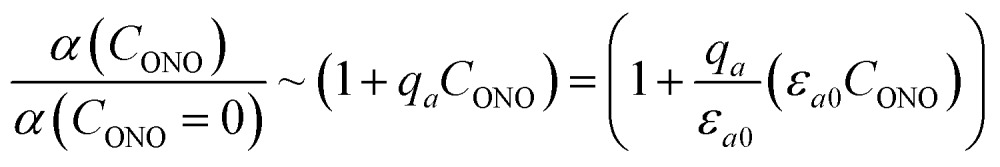
that corresponds to the effect of strain along axis *a* which results from the transformation and equals to *ε*_*a*0_*C*_ONO_. The value of the coefficient *q*_*a*_/*ε*_*a*0_, 32 ± 3, is comparable to that of *q*_*T*_. The difference in the values of *q*_*T*_ and *q*_*a*_/*ε*_*a*0_ may be due to the difference in anisotropy of the deformation tensors corresponding to strain caused by thermal expansion and by the photoisomerization.^57^ This value of *q*_*a*_ indicates that the quantum yield of photoisomerization decreases approximately twice by the time the reaction is completed as compared to its onset.

**Fig. 9:**
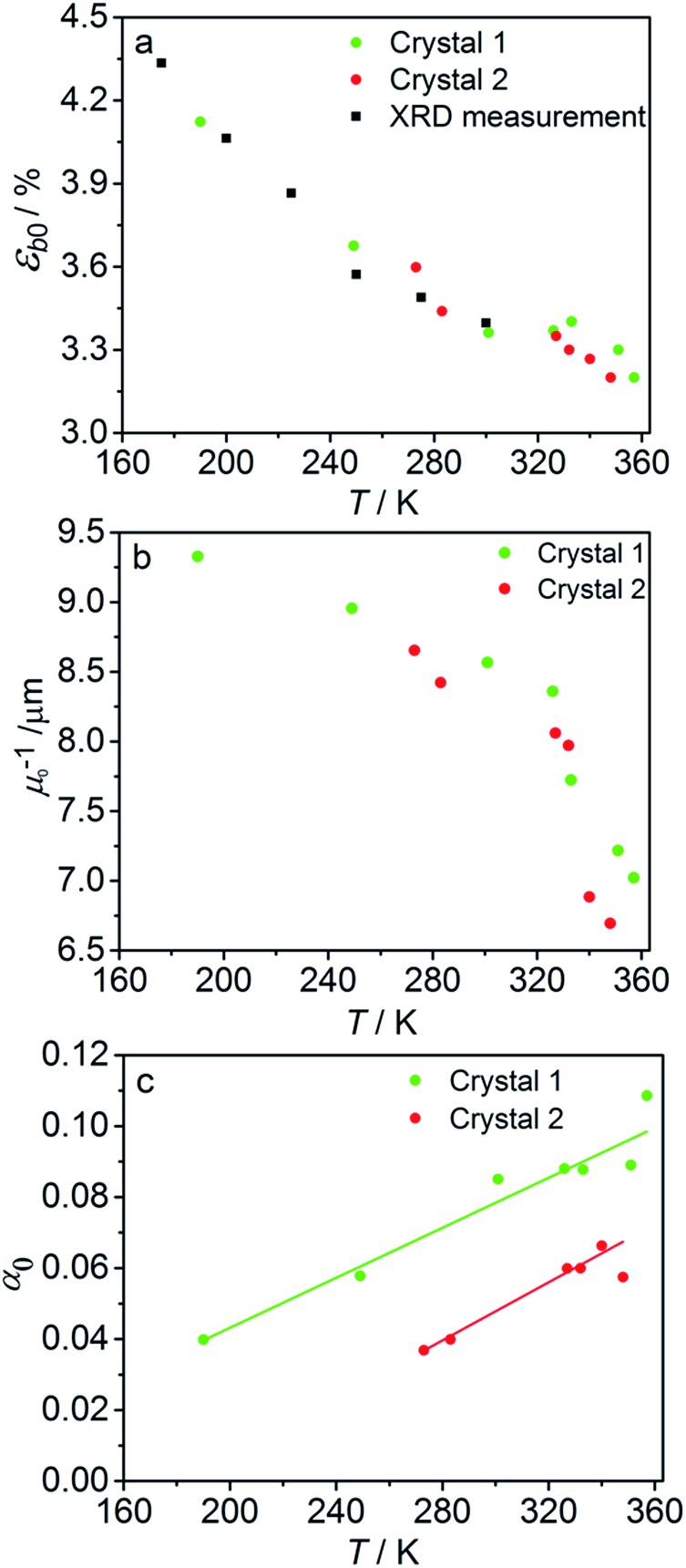
Temperature dependence of the strongest deformation (a), characteristic penetration depth (b), and quantum yield (c) obtained by fitting the experimental data for two different crystals to the curves simulated with the kinetic model given by eqn (15) and (16). For comparison, data on *ε*_*b*0_ obtained from single crystal X-ray diffraction experiment are also plotted in panel (a).

The fitted temperature dependence of the strongest deformation (*ε*_*b*0_), the characteristic penetration depth *μ*_0_^–1^, and the quantum yield for two different crystals are compared in Fig. 9. The values of *ε*_*b*0_ for the two different crystals are consistent with each other (Fig. 9a). Moreover, their deviation from the results from X-ray diffraction experiments does not exceed 0.1% across the whole temperature range. The values of the absorption coefficient *μ*_0_ are also similar (Fig. 9b), and are more consistent relative to *μ* predicted using a less sophisticated model that neglects the difference in the absorption of the two isomers (Fig. 7b). The variation of *μ*_0_ with temperature confirms that the nitro form 1-*N* absorbs light stronger than the nitrito form 1-*O* (seen in the initial stages of the forward reaction 1-*N* → 1-*O* at *T* > 330 K).

The temperature dependence of the quantum yield estimated using the advanced model (Fig. 9c) qualitatively resembles that obtained using the simplified model (Fig. 7c), although the quantitative differences are apparent. The thermal variation of the quantum yield can be directly compared to that described by eqn (17). The corresponding correlation coefficients *q*_*T*_ are slightly different when estimated for different crystals. The values for the two crystals for which the data are plotted in Fig. 9, 65 ± 4 and 108 ± 5, are comparable to the value 55 ± 5 reported in ref. 57. The discrepancy between different crystals may be a consequence of crystal imperfections, as it is normally anticipated for real solids. This difference could also be related to small variations in the irradiation conditions between different experiments (see ESI Note 9†). Assuming identical quantum yields but different photoisomerization rates (due to different light intensity) for different crystals, the experimental data for different crystals imply nearly 1.7 times difference in light intensity (compare with the estimated factor of 2 in the ESI Note 9†), giving an average value of *q*_*T*_ = 70 ± 7.

The preceding analysis confirms that all parameters and their temperature dependence are reasonably modeled by analysis of the temperature-dependent macroscopic strain using the advanced model, and can be assessed against complementary experimental data. The difference in the values of *α*_0_ obtained from different crystals in particular illustrates the importance of using the same crystal in all measurements. As outlined above, this is possible only if the photoreaction is reversible and the crystal is capable of multiple photothermal reaction cycles.

#### 2.7.5. The effect of chromophore orientation with respect to light

**Fig. 10:**
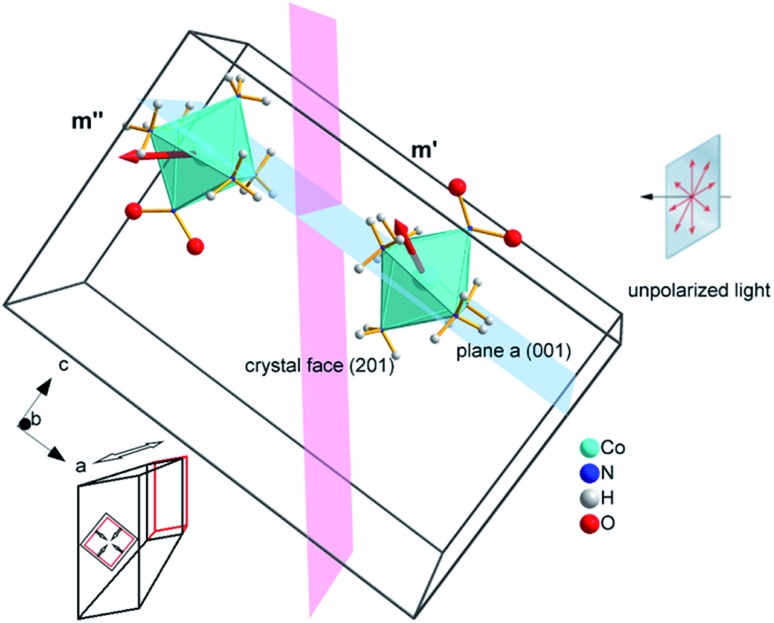
Two types of complex cations in the crystal of 1-*N* with different orientations of the transition dipole moment with respect to the direction of light flux irradiating the crystal normal to face (201) (shown as a plane colored pink). The glide *a* relating the two complex cations is shown as a plane colored blue. Cl^–^ and NO_3_^–^ anions as well as two additional complex cations in the unit cell are not shown for clarity. Directions of principal strains in the crystal are schematically shown in the bottom left corner.

The fits can be additionally improved by taking into account the difference in light absorption between the different chromophores in the crystal (in case of the reaction studied here, the complex cations). In the structure of 1-*N*, the complex cations [Co(NH_3_)_5_*N*O_2_]^2+^ are oriented in four different dispositions with respect to the crystallographic axes, and two pairs with different orientations are related by inversion. The inversion does not change the interaction of a molecule with light; thus, only two types of cations interact with light. The orientations of these two different species are related by reflection over a glide plane (glide *a* in the space group of the crystal structure) normal to the crystallographic direction *c* (normal to the long crystal axis and to axis *a* in crystal cross-section, Fig. 10).

If the irradiation normal to the crystal axis was directed along the *a* axis, there would not be any difference in the interaction of these two types of cations with light. However, the irradiated face of the crystal is (201) (or the symmetrically equivalent (201)) so that the orientations of the transition dipole moments of the two cations with respect to the light direction normal to the axial crystal face are different. The interaction of the two different types of complex cations [Co(NH_3_)_5_*N*O_2_]^2+^ can be described by two different absorption coefficients, *μ*′_0_ and *μ*′′_0_. The initial absorption coefficient of the crystal is defined by the average, (*μ*′_0_ + *μ*′′_0_)/2. On irradiation, the cations having larger absorption coefficient are transformed into the nitrito isomer faster. This results in effective decrease in the total absorption by the remaining nitro isomers. This effect can explain the dependence of *μ*_0_ on the temperature (Fig. 9b; see also ESI Note 10†).

The light source used in the experiments was unpolarized, and can be considered a combination of uniformly distributed linearly polarized waves. The angular density of these linearly polarized waves is *i*_0_(*θ*_p_) = *I*_0_/2π. As a result of light absorption and 1-*N* → 1-*O* phototransformation, the initial uniform angular density will change to the time-dependent non-uniform distribution *i*(*θ*_p_,*x*,*t*) inside the crystal. The absorption eqn (15) then becomes19
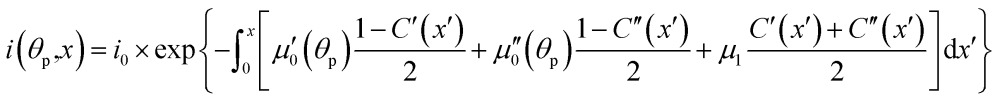
where *C*′ and *C*′′ are the transformation degrees of the two different types of nitro isomers. The absorption by the two different types of nitro isomers can be represented as20
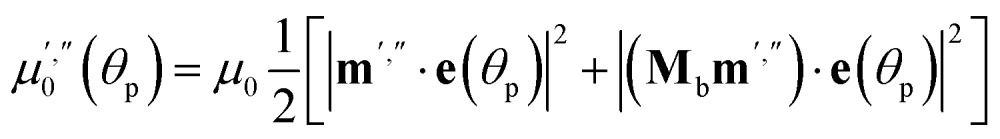
where **e** is the direction of the polarization vector with the polarization angle *θ*_p_, **m′** and **m′′** are the unit vectors along the directions of the transition dipole moments of these species, *μ*_0_ is the absorption coefficient of the nitro isomer oriented so that the transition dipole moment is lying along the wave polarization vector.

Two terms in the eqn (20) are related to the fact that the complex cation is located at the mirror plane normal to the long crystal axis (axis *b*). **M**_b_ is the corresponding symmetry operator. The form of the eqn (20) assumes that the direction of incident light is strictly normal to the long crystal axis (axis *b*). In general case, if a crystal is inclined with respect to light, corresponding rotation operators should be applied to both transition dipole moments (ESI Note 11†). The components of **m′** and **m′′** are defined in the laboratory coordinate system in which the light is parallel to the *x* direction, the crystal axis is lying along *y* with the face (201) normal to the light beam. Thus, these vectors can be defined with the following equations

21 **m′** = **C**_b_(*φ*_0_)**m**_0_**m′′** = **C**_b_(*φ*_0_)**M**_c_**m**_0_

where **C**_b_ is the operator of rotation around the *b* axis at the angle between the *a* axis and the normal vector to (201) face, *φ*_0_ = 0.652, **M**_c_ is the operator of the mirror reflection normal to the *c* axis (symmetry connection between the two complex cations), **m**_0_ is the direction of the transition dipole moment of one of the cations in the crystal coordinate system. The kinetic equation given by eqn (16) transforms into the system of two equations for two types of the nitro cations22
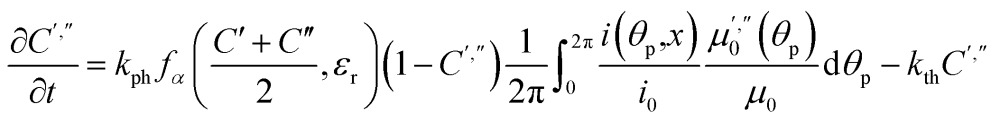
where the other coefficients are defined above.

**Fig. 11:**
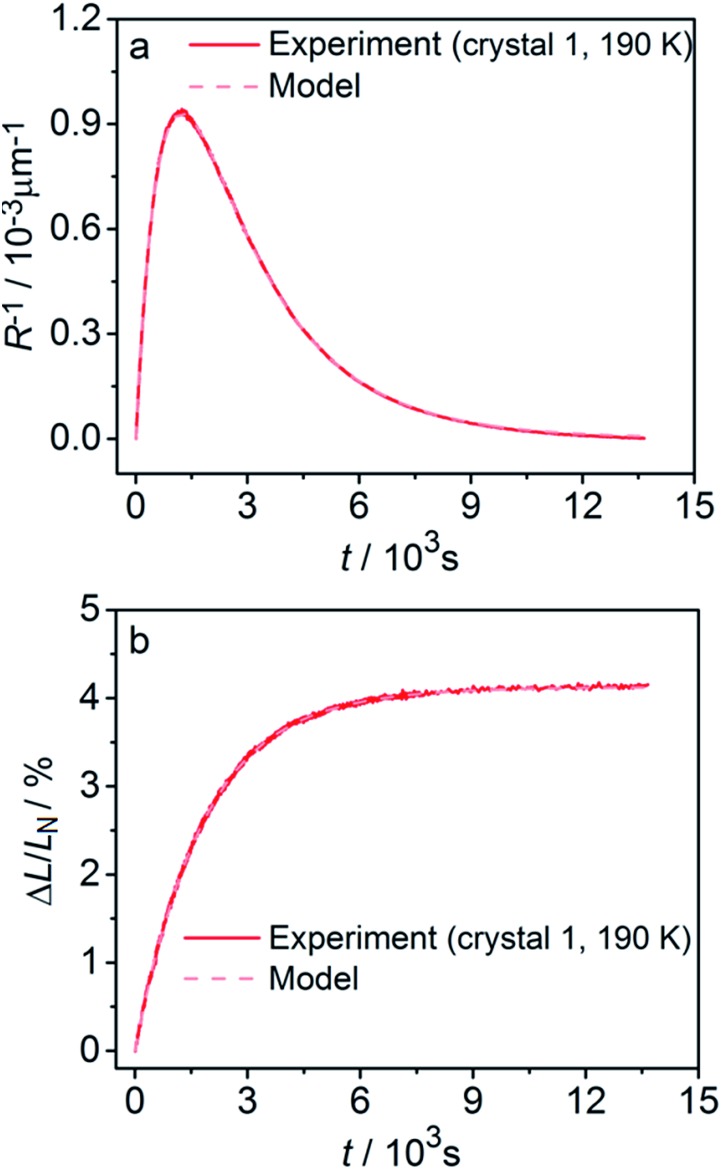
Fitting the experimental curvature (a) and relative elongation (b) with functions derived from eqn (19)–(22).

Fitting experimental data using this model (eqn (19)–(22)) is computationally demanding. The results depend on the exact orientation of the crystal with respect to light, which may vary between different experiments and different crystals. As an illustration we present in Fig. 11 the result of single fitting for experiment carried at 190 K on one single crystal. The residuals of the fit are solely defined by the noise in the experimental data. The fitting parameters are *ε*_*b*0_ = 0.04127 ± 1 × 10^–5^, *q*_*a*_ = –0.628 ± 0.005, *α*_0_ = 0.0394 ± 1 × 10^–4^, *μ*_0_^–1^ = 3.6 ± 0.2 μm, *μ*_1_^–1^ = 34.6 ± 0.2 μm. The transition dipole moment direction in the crystal system is defined by a vector **m** = (–0.505, –0.77, 0.389) ± (0.13, 0.11, 0.13).

The values of the elongation *ε*_*b*0_, the feedback coefficient *q*_*a*_, the estimated quantum yield *α*_0_ and the averaged absorption coefficient for the 1-*O* isomer obtained using this model are close to the values obtained using eqn (15) and (16). The values of the characteristic depth of light absorption (averaged over all polarization planes in the incident light) for the two orientations of the complex cations are *μ*′_0__*θ*_p__^–1^ = 7.5 ± 0.5 μm and *μ*′′_0__*θ*_p__^–1^ = 12.3 ± 0.8 μm. This result means that on average the cations in a more favorable orientation must isomerize 65% faster. The average absorption of the unpolarized light by the reactant isomer 1-*N* in the crystal can be estimated from the obtained transition moment direction and the value of *μ*_0_ to give the characteristic penetration depth *x*_0_ = 2/*μ*′_0_ + *μ*′′_0__*θ*_p__ = 9.3 ± 0.6 μm. This value is close to all previous estimations of this parameter.

The above values can be used to explain the results in Fig. 9b. At *T* > 330 K, that is, at temperatures that favor low conversion 1-*N* → 1-*O*, the main contribution to the reaction comes from the cations with higher absorption coefficient (characteristic light penetration depth ∼ 7.5 μm). At *T* < 330 K, the reaction proceeds almost completely. Both types of cations with different orientations are involved so that the characteristic light absorption is close to the average of the values corresponding to each of the orientations (characteristic light penetration depth ∼ 9.3 μm). Another important parameter which can be calculated from the determined absorption parameters is the molar extinction coefficient corresponding to the absorption, averaged over all possible space orientations of the complex cation, as it appears in a solution. Its calculated value, *μ*_0_*N*_a_*v*_0_/(3 ln(10)) = 61 ± 3 L mol^–1^ cm^–1^ is in agreement with the reported value of absorption by the complex cation at the absorption maximum near 460 nm.^48^,^50^,^51^

### 2.8. Limitations of the general model for photoinduced bending of crystals

The general model elaborated above makes explicit assumptions related to the compound, the crystal and the transformation that must be fulfilled in order to be directly applicable to other photobendable crystals. In cases when a specific requirement is not met the model can be used as a reasonable approximation or it can be accordingly modified. The transformation must be of a single-crystal-to-single-crystal type; only then the transformation extent is unambiguously related to the strain caused by the transformation, and as a consequence, the macroscopic mechanical response (bending or elongation) can be related to the kinetic parameters of the reaction. If this requirement is not met because of, for example, increased mosaicity or other imperfections, the relation between the transformation extent and mechanical strains can be considered in the mean-field approximation, by analogy with mechanical properties of polycrystalline materials. The crystals must have a needle-like shape (aspect ratio less than 1 : 10), as this is a prerequisite to measure the strain that develops upon bending with sufficient precision so that it can be used in quantitative analysis. The optimal strains are in the range of several percent. Too small deformations (<0.1%) result in macroscopic strain that is too small to be measured reliably, whereas too large deformations (>10%) will likely induce relaxation of mechanical stress *via* plastic relaxation, so that there is no direct relation between the macroscopic strain and the microscopic deformation.

There are also restrictions on the kind of mechanical response which should not include deformation modes like twisting of crystals and should be restricted by pure uniform bending. The case of twisting can still be modelled.^1^ However, it introduces serious difficulties in experimental techniques (measurement of deformation characteristics of curvature, torsion and elongation or contraction) and in theoretical treatment (complicated change of the irradiation conditions in course of transformation). Whether the twist appears or not, is defined by certain symmetry features of the strain and strength tensors specified earlier.^1^ The optimal thickness of the crystals is comparable with that of the characteristic absorption depth corresponding to the wavelength of light inducing the phototransformation. For much thicker crystals (*h*/*x*_0_ > 10) relaxation of the mechanical stresses will occur in the surface layer of the irradiated crystal. In such case the reaction can be studied only up to a small extent of transformation (the experimental setup can be modified using reflection of the laser beam from a mirror fixed at the end of the crystal^52^). The relatively low coefficient of light absorption (typically, the extinction coefficient less than ∼1000 L mol^–1^ cm^–1^) is directly related to the restriction on the crystal thickness. Strong light absorption requires manipulation of very thin (submicron crystals), which is technically challenging. Finally, the model requires that the phototransformation is reversible and the crystal does not change its properties after multiple cycles of bending and straightening. This provides the possibility to use the same crystal in multiple measurements under a variety of conditions. This strategy increases the reliability and the reproducibility of measurements, and excludes the data scatter related to factors that pertain to the imperfections of individual real crystals.

In cases of not strict fulfilment of the limitations the quantification of crystal deformation with the model still can be used as an instrument for systematic estimation of main quantities defining the phenomenon of photoinduced bending like apparent transformation rate constants and light penetration depth which can be treated as a characteristic size of the transformation non-uniformity in the thickness of crystals. Moreover, the very fact of the experiment deviating from the model can indicate certain features of the process that may be not obvious from the first sight (such as the contribution of plastic deformation to elastic bending, the existence of shear components of stress, incomplete reversibility, *etc.*). It is also to note, that the conditions imposed on the phototransformation, in order to enable the best applicability of the model to describe the experimental observations, are the same that are required for the crystals undergoing this transformation to be used in a photomechanical device. Thus, testing the applicability of the model to the experimental behaviour of photobendable crystals can be used to evaluate the potential of these crystals as supramolecular actuators or sensors.

## 3. Experimental details

### 3.1. Measurement of the uniform crystal elongation

Six crystals of [Co(NH_3_)_5_*N*O_2_]Cl(NO_3_) ranging between 450 μm and 1700 μm in length and between 20 μm to 30 μm in thickness were studied. Prior to the measurement every crystal was attached by one end to a glass plate with a small drop of glue so that the crystal was parallel to the glass surface. The crystal was irradiated in two steps to prevent possible cracking because of non-uniformity of transformation at intermediate stages. Initially the crystal was irradiated with 1 W green LED (520 nm) during 15 hours at room temperature and subsequently with 1 W blue LED (465 nm) during 3 hours at –20 °C. Then the glass with the crystal was installed inside the thermally controlled camera held at a certain temperature and mounted to the stage of optical transmitting microscope. The camera was made from a massive copper cylinder with internal controlled heater and optical window (Fig. 1a). A period of 1 min was allowed for the installed plate to reach the desired temperature, and the displacement of the free crystal end relative to the scale on the glass plate was monitored as a function of time.

### 3.2. Measurement of the photoinduced crystal bending

A specially designed thermal camera for these measurements is shown in Fig. 1b. The crystal was attached to the glass capillary and positioned inside the camera in the spot of a condensed 3 W blue LED (465 nm) normally to the light beam. Double windows with external air-blowing system were used to prevent condensation of water vapor and icing at low temperatures in all three positions (irradiation, lamp and microscope). The approximate diameter of the light spot around crystal was ∼1 cm. The overall photon flux coming through a 1 cm-aperture was measured with an optical powermeter outside the camera with identical geometry as in the experiment to produce irradiation power 0.05 ± 0.005 W. The measured irradiation power corresponds to the average photon flux density in the light spot *I*_0_ = (1.5 ± 0.15) × 10^17^ photons/(s cm^2^). The measured photon flux density can be used as upper estimate of the real value corresponding to the irradiation conditions for crystals installed in the camera. The deviations from this value are related to uncontrolled transmission of the inlet window and non-uniformity of the flux density over the spot. The temperature of the crystal was maintained with a stream of gas and measured with two thermocouples, the first installed at the gas tube outlet, and the second affixed downstream, just behind the crystal. Dry nitrogen evaporated from a liquid nitrogen tank and supplied through an all-metal gas line was used to dehumidify the camera. A halogen lamp installed into the tank was used to regulate the gas flow. The total gas flow was divided into two parts, one of which was heated to a controlled temperature with a tube heater, while the second one was cooled down in a coil heat exchanger deep in another liquid nitrogen tank. Changing the heater temperature and hot/cold nitrogen flow ratio allowed us to maintain the crystals at a selected temperature in the range from 120 K to 370 K, with the difference between the two thermocouples not exceeding 3 K. Temperature of the downstream thermocouple was taken as the sample temperature.

The stationary bending experiment (Section 2.5) was conducted as follows. With the irradiation switched on and the temperature set to a desired value we were waiting for the crystal to stop its deformation. Then we took a photograph of the crystal to measure the deformation reached. We changed the temperature, so that the crystal shape was changing for some time and waited until the deformation stopped, and a photograph was taken again. Thus, after repeating this procedure several times we obtained the dependence of stationary bending on temperature.

Every isothermal bending/unbending experiment (Section 2.7.1) was preceded with thermal annealing of crystals at 360 K for one hour in the darkness. Then the desired temperature was reached and stabilized during another hour. After this procedure the irradiation was switched on and the kinetics was measured. The bending/unbending process was registered as time-lapse photographs. The image series was analyzed (Fig. S1 in the ESI†) with a software developed as a specialized plugin for ImageJ^74^ (the plugin “Bending Crystal Track” is available at ImageJ website ; http://imagej.net/PhotoBend). The curvature and length change were calculated from coordinates of three points corresponding to two ends and the center of the crystal defined by the image template matching technique. Knowing the three values equal to the length of chords (*H* – the chord connecting the two ends of the crystal, *H*_1_ and *H*_2_ – the chords connecting the end of the crystal with its middle, Fig. S1 in the ESI†), the angle corresponding to the crystal arc is calculated:23
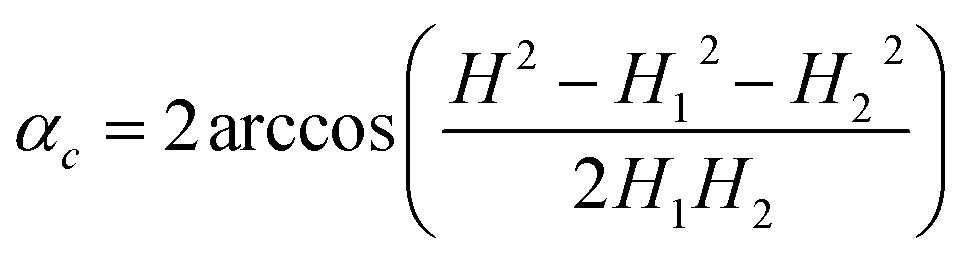



The value of this angle was used to calculate the crystal curvature24
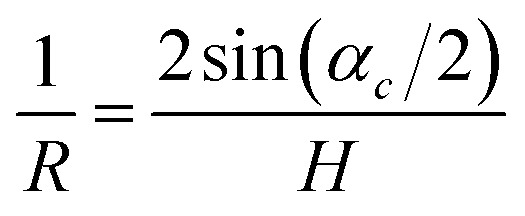
and its length

25 *L* = *Rα*_*c*_

The complete set of measurements was carried out with two crystals with slightly different geometrical parameters. The data discussed in this paper correspond to crystals labeled “crystal 1” (706 μm long and 23 μm thick) and “crystal 2” (644 μm long and 20 μm thick). We show the experimental data for the crystal 1 in the main text and for the crystal 2 in ESI.† Fig. 9 shows the comparison of resulting parameters obtained for both crystals: *ε* and *μ* are the same, while quantum yields are different (presumably, because of different irradiation intensity because of different installation inside the camera, or different crystal imperfections, *e.g.* different scattering by the surface).

### 3.3. X-ray diffraction determination of the strain related to photochemical and thermal reaction

X-ray diffraction data for solid solutions of 1-*N*/1-*O* were collected at beamline ID15B in ESRF (Grenoble, France; experiment CH-4908) using a parallel monochromatic X-ray beam (*E* = 30 keV, *λ* = 0.4109 Å). Single-crystal data were collected by a vertical-acting *ω*-axis rotation with an integrated step scan of 0.5° and a counting time of 1 s per frame. A MAR555 flat-panel detector was used to record diffraction intensities. Typical data collection time for one point was ∼15 min. Cell determination and data integration were performed using the CrysAlisPro software.^75^ Refinement was carried out with SHELXL-2016 (ref. 76) using ShelXle as the GUI.^77^ Atomic parameters for all heavy atoms were refined anisotropically without any restraints. The hydrogen atom positions of NH_3_ were constrained to an idealized disordered model with two equal positions at an angle of 60°. The site occupancy factor of 1-*O* was refined as a second free variable in SHELXL-2016 adding to full occupancy with 1-*N*.

To obtain 1-*O*, a crystal of 1-*N* was irradiated by focused green LED light for 12 hours, followed by irradiation by focused LED blue light for 1 hour (experiment 2). Very low electron density peak corresponding to traces of 1-*N* were detected, however a reliable refinement of corresponding site occupancy factors was not possible and this component was not included in the refinement. Mixed crystals with intermediate concentrations of both isomers (experiments 3–5) were obtained by irradiation and data collection at ∼2 h intervals at 295 K. In the high-temperature experiments (experiments 6–9) the crystal was additionally annealed at 333 K for 2 minutes. Before experiment 10, the crystal was annealed at 343 K for 50 minutes to obtain 1-*N*. Before the experiment 11, 1-*N* was irradiated by focused blue LED light for 2 hours to obtain individual 1-*O*, and subsequently annealed for 7 minutes at 333 K before experiment 12. The details of the data collection and refinement details are summarized in Table S2.† Complete structural data were deposited in the CSD.^78^

The temperature dependence of cell parameters of 1-*N* and 1-*O* was measured using STOE IPDS II diffractometer with an image-plate detector, MoKα radiation and an Oxford Cryostream cooling device. Diffraction data for cell refinement were collected at 300, 275, 250, 225, 200 and 175 K. Reflection measurements for 0.40 × 0.03 × 0.03 crystal of 1-*O* were performed during continuous irradiation by focused light of 3 W-blue LED. The same crystal was annealed for 1 h at 353 K to obtain 1-*N* isomer and used for further cell parameter measurements. The software X-AREA^79^ was used for data collection and cell refinement. The details on data collection and cell refinement are summarized in Table S3.†

## Conclusions

There is an increasing number of reports of photomechanical effects of crystals, however, these effects are rarely explained beyond the mere observation that crystals can bend when exposed to light of appropriate wavelength. These photomechanical effects, however, conceal an exciting solid-state photochemistry, that warrants robust and sophisticated mathematical models to extract valuable information. Careful measurements of changes in crystal curvature and elongation due to photochemical transformation at different temperatures can provide information on the kinetic parameters (the reaction constants of the direct photochemical reaction and the reverse thermal reaction, and the activation energies of these reactions). Additional parameters, such as the quantum yield, reaction feedback parameters, and even the orientation-dependent absorption coefficients of the chemical species in the crystal can be extracted. Many of these static and kinetic parameters are hardly accessible with other, classical experimental methods, such as spectroscopy or X-ray diffraction.

In this work, we illustrate the applicability of this approach on a selected example of a solid-state reaction system that undergoes photoinduced reversible linkage isomerization [Co(NH_3_)_5_*N*O_2_]Cl(NO_3_) ↔ [Co(NH_3_)_5_*O*NO]Cl(NO_3_). The model elaborated here, however, is general and within the assumptions made in respect to the compound, the crystal and the transformation (Section 2.11), it can be applied to a wide variety of other phototransformations in crystals. The method for analysis of the kinetics of phototransformation by measuring the macroscopic strain that develops during elastic bending of a needle-shaped crystal is practically self-sufficient and does require very little additional information. It provides that following parameters: (a) the rate constant of the thermal reaction *k*_th_ and its dependence on the temperature, (b) the rate constant of the photochemical reaction *k*_ph_ and the quantum yield *α* (for known light intensity), (c) the dependence of *α* on the temperature and transformation extent, (d) the light absorption coefficients for the reactant and the reaction product, and (e) the orientation of the vector of the dipole moment of the electronic transition in the crystal structure.

The only information required from complementary X-ray diffraction experiments is the dependence of lattice strain on the extent of transformation. In fact, the maximal strain achieved at complete transformation can be estimated from measuring the crystal deformation (without XRD experiments). In some selected cases, when strain is linearly related to the transformation degree, and there is a reverse thermal reaction described by the first-order kinetic law, the measurement of the kinetics of mechanical response may be sufficient even without the XRD. However, the XRD analysis gives the unambiguous information on the relation between macroscopic crystal deformation and the degree of photochemical transformation. In the present study, the discrepancy between the estimated lattice strain based solely on the measurements of the macroscopic crystal deformation and on precise single-crystal X-ray diffraction data does not exceed 0.1%. The uncertainty in the measurement of the strain is determined by two factors: the resolution of the analyzed images (the uncertainty with the method used here is about 1 pixel) and possible errors in the analysis of the crystal geometry because the ratio of the crystal thickness to its length is not infinitely small. Taking into account the two factors in the case described in this work gives an estimate in the error of the measured strain of about 0.1%. The method described here is the most comprehensive to date available method for kinetic and kinematic analysis of photobendable crystals, and is the only available means to obtain parameters that are specific for the interaction of light with the crystal and the photochemical reaction in the crystalline state. It provides information on both the average transformation degree and the spatial distribution of the reaction product through the crystal depth, in contrast with the other techniques that provide only average values.

## Conflicts of interest

There are no conflicts to declare.

## Supplementary Material

Supplementary information

Crystal structure data
