# Supplementary material for: Quantification of photoinduced bending of dynamic molecular crystals: from macroscopic strain to kinetic constants and activation energies
Source: Chem Sci. 2018 Jan 22;9(8):2319–35. doi: 10.1039/c7sc04863g (PMC5903420; doi:10.1039/c7sc04863g)
Supplement: Supplementary file 1 [file SC-009-C7SC04863G-s001.pdf]

Electronic Supplementary Material (ESI) for Chemical Science.
This journal is © The Royal Society of Chemistry 2018

SUPPORTING INFORMATION

Quantification of Photoinduced Bending of Dynamic Molecular Crystals: From Macroscopic Strain to Kinetic Constants and Activation Energies

Stanislav Chizhik,[1,2,*] Anatoly Sidelnikov,[1,2] Boris Zakharov,[1,2] Pančе Naumov,[3] Elena Boldyreva[1,2]

[1]*Institute of Solid State Chemistry and Mechanochemistry, Siberain Branch of Russian Academy of Sciences, ul. Kutateladze, 18, Novosibirsk 620128 Russian Federation*

[2]*Novosibirsk State University, ul. Pirogova, 2, Novosibirsk 630090 Russian Federation*

[3]*New York University Abu Dhabi, P.O. Box 129188, Abu Dhabi, United Arab Emirates*

Contents:

Supporting notes Note 1 – Note 11 S2

Figure S1. Screen snapshot of the software used to record the crystal bending showing the procedure for measurement of the crystal curvature and length. S4

Figure S2. Dependencies of cell parameters *a*, *b*, *c* and cell volume *V* on the content of 1-*O* (nitrito) isomer in the crystals of $[Co(NH_3)_5NO_2]Cl(NO_3)$. S4

Figure S3. Temperature dependencies of the relative changes of the cell parameters *a* (a), *b* (b), *c* (c) caused by complete phototransformation from 1-*N* to 1-*O*. S5

Figure S4. Crystals attached to the capillary and used in the bending experiments: crystal 1 (a), crystal 2 (b) S5

Figure S5. The kinetics of changing the curvature (a) and the length (b) of crystal 2 (to compare with those for crystal 1 see Figures 6ab). Experimental data (solid lines) and quantitative description of the kinetics using the model with constant coefficients (dashed lines) described by eqs 4, 8 and 11. S6

Figure S6. The kinetics of changing the curvature (a) and the length (b) of crystal 2 (solid lines) and quantitative description of the kinetics (dashed lines) using a model with the coefficients depending on the transformation degree described by eqs 15-16. S7

Table S1. Combination of crystal samples and temperatures used to verify the reproducibility of the measurements under conditions of uniform both-sides irradiation of single crystal of 1-*N* and thermal reversion of the resulting 1-*O*. S8

Table S2. Data collection and refinement details for solid solutions $[Co(NH_3)_5NO_2]ClNO_3/[Co(NH_3)_5ONO]ClNO_3$. S9

Table S3. Variable-temperature cell refinement for $[Co(NH_3)_5ONO]ClNO_3$ and $[Co(NH_3)_5NO_2]ClNO_3$. S12

Table S4. Dependence of the thermal isomerization rate constant, k_{th}, on temperature. S13

Supporting notes

Note 1. The ratios >10 result in high level of stress in the transformed layer leading to mechanical relaxation processes such as plastic deformation or fracture. The relaxation changes all relevant properties of the crystal drastically and abruptly, and does not provide reproducible, meaningful quantitative results.[1,53]

Note 2. This reaction has already been used by us to relate the quantum yield of photoisomerization and the mechanical stress that originates from external loading[52] or thermal expansion.[57]

Note 3. The characteristic light penetration depth x_0 is calculated as the reciprocal of the absorption coefficient μ in the Beer-Lambert law. The absorption coefficient is connected with the molar extinction coefficient ε by $\mu = \varepsilon C \ln(10)$, where C is the molar concentration of the absorbing substance.

Note 4. A thermal equilibrium between 1-*O* and 1-*N* was assumed in some previous works.[59,66] In such case, the measured value of the thermal isomerization rate constant would be equal to the sum of the two rate constants, those of the forward (nitrito-nitro) and backward (nitro-nitrito) thermal reactions, $k_{\mathrm{th}} = k_{\mathrm{th}}^{\mathrm{f}} + k_{\mathrm{th}}^{\mathrm{b}}$. The relation between the two thermal constants is defined by the equilibrium constant K and the corresponding Gibbs energy change ΔG, with $k_{\mathrm{th}}^{\mathrm{f}} / k_{\mathrm{th}}^{\mathrm{b}} = K = \exp(-\Delta G/RT)$. For 1-*O* the value of the equilibrium constant is not known. For the compounds with the same complex cation, $[Co(NH_3)_5ONO]X_2$ (X = Cl^-, Br^-), the ΔG values are between –6 kJ/mol and –13 kJ/mol, which gives $k_{\mathrm{th}}^{\mathrm{b}}$ that does not exceed ~10% of the $k_{\mathrm{th}}^{\mathrm{f}}$ at the highest temperature used in this work (T = 360 K). The single crystal XRD study of 1-N crystals heated up to T = 393 K did not reveal the presence of any traces of the 1-*O* in the crystal.[57] This result refutes the hypothesis of a thermal equilibrium between the two isomers in the crystal. Should the 1-*O* isomer still be present, its amount does not exceed 5% (mol). The corresponding value of ΔG at this temperature then should not be greater than –10 kJ/mol. In the present study, where the temperatures did not exceed 363 K, the thermal nitro-nitrito isomerization can be safely neglected, and the heating can be assumed to result exclusively in nitrito-nitro 1-*O* → 1-*N* transformation.

Note 5. The average residuals did not exceed 2×10^{-2} cm^{-1}, that is, less than 1% of general magnitude of the curvature in the experiments.

Note 6. Equation 4 was obtained for the simplest possible assumption that the absorption coefficient μ is constant and the photoreaction constant k_{ph} does not depend on the transformation degree.[1] The same equation also holds in more general cases, when α and μ are substituted with the corresponding functions of the transformation degree (and, possibly, other parameters, such as temperature or mechanical stress). The additional difference in more general cases of non-constant values will be in introducing two different absorption coefficients in this equation: μ appearing as a separate coefficient in Eq. 4 will be the total absorption coefficient in the substance (including absorption by the reactant and by the product), while μ in the rate constant k_{ph} will correspond to the absorption contributed by reactant only. The analysis of the stationary crystal curvature with the approximate eq 4 is reasonable in order to estimate the temperature dependence of the relation between the constants $k_{\mathrm{th}}/k_{\mathrm{ph}}$ as it gives the most impact in the resulting stationary curvature due to high temperature dependence of the k_{th}. Another implication in this analysis is usage of crystals not strictly satisfying to the $\mu h \ll 1$ limiting case. But a numerical simulation shows that the eq 4 remains qualitatively correct up to $\mu h \sim 3$. The result mostly influenced by the deviation from the thin crystal limit is connected with definite shift of the maximum position to the lower values of $k_{\mathrm{th}}/k_{\mathrm{ph}}$.

Note 7. The reaction 1-*N* → 1-*O* results not only in expansion of the crystal along its longest axis but also in compression normal to that axis (along axis ***a***; the maximum strain is –2.4% after complete transformation, see Fig. S3 in the SI). This compression is known to lower the quantum yield.[57] As the maximum transformation extent decreases with increasing temperature because of the increased contribution of the reverse reaction 1-*N* ← 1-*O*, the lattice contraction along *a* decreases as well, thus contributing to higher quantum yield. Taken together, these results show that both the thermal expansion and the decrease in the

average transformation extent at higher temperatures contribute to higher quantum yield of photoisomerization.

Note 8. The absorbance of the nitrito form is thus estimated to be 5 to 7 times lower than that of the nitro-isomer. This difference is larger, than that measured for the two isomers in aqueous solutions. However, absorption in solution may differ from absorption in the solid state. There is presently no direct experimental information on the UV-Vis absorption of the nitrito-isomer of $[Co(NH_3)_5ONO]Cl(NO_3)$ in the solid state to assess the result. Additionally, the absorption in crystals depends not only on the oscillator strength of the corresponding electronic transition, but also on the crystallographic orientation of the absorbing species and the polarization plane of the irradiation causing the excitation. So, the lower predicted absorption of nitrito form as compared to solution data can be a consequence of a specific relative orientation of incident light and the dipole moment of corresponding transition in the nitrito-isomer in the irradiated crystals.

Note 9. The value of I_0 is the highest estimated value of the used light source. It has been measured outside of the experimental setup (see the Experimental details). Real photon flux on the crystal installed inside the measurement camera can be somewhat lower because of the intensity loss on the camera window and because of inevitable irreproducibility of the crystal installation relative the light spot inside the camera. We assume that up to two-fold total photon flux density decrease may result from the different crystal installations comparing to the initial value of I_0.

Note 10. The same reasoning can be given for the interaction of differently oriented nitrito-isomers with light. However, since the nitrito-isomers in the crystal are no longer involved into the phototransformation, we have assumed for the sake of simplicity the light absorption by all the nitrito-isomers to be the same and isotropic.

Note 11. We do not add any corrections taking into account the possible effect of changing the angle between the crystal and the light beam caused by crystal bending. This effect would not exceed $\pm 11^{\circ}$ for the present experimental data (the refraction inside the crystal should be accounted for, to make this estimation). This deflection is supposedly not larger than deviations caused by thermal vibration in the lattice or by the transformation itself (the nitrito species are acting as point defects causing local lattice distortions).

Supporting figures

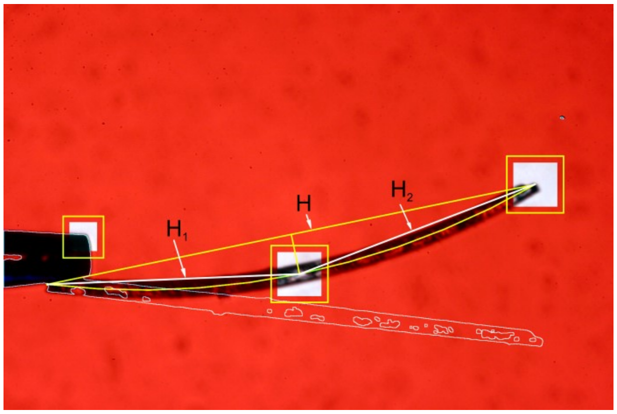

Figure S1. Screen snapshot of the software used to record the crystal bending showing the procedure for measurement of the crystal curvature and length.

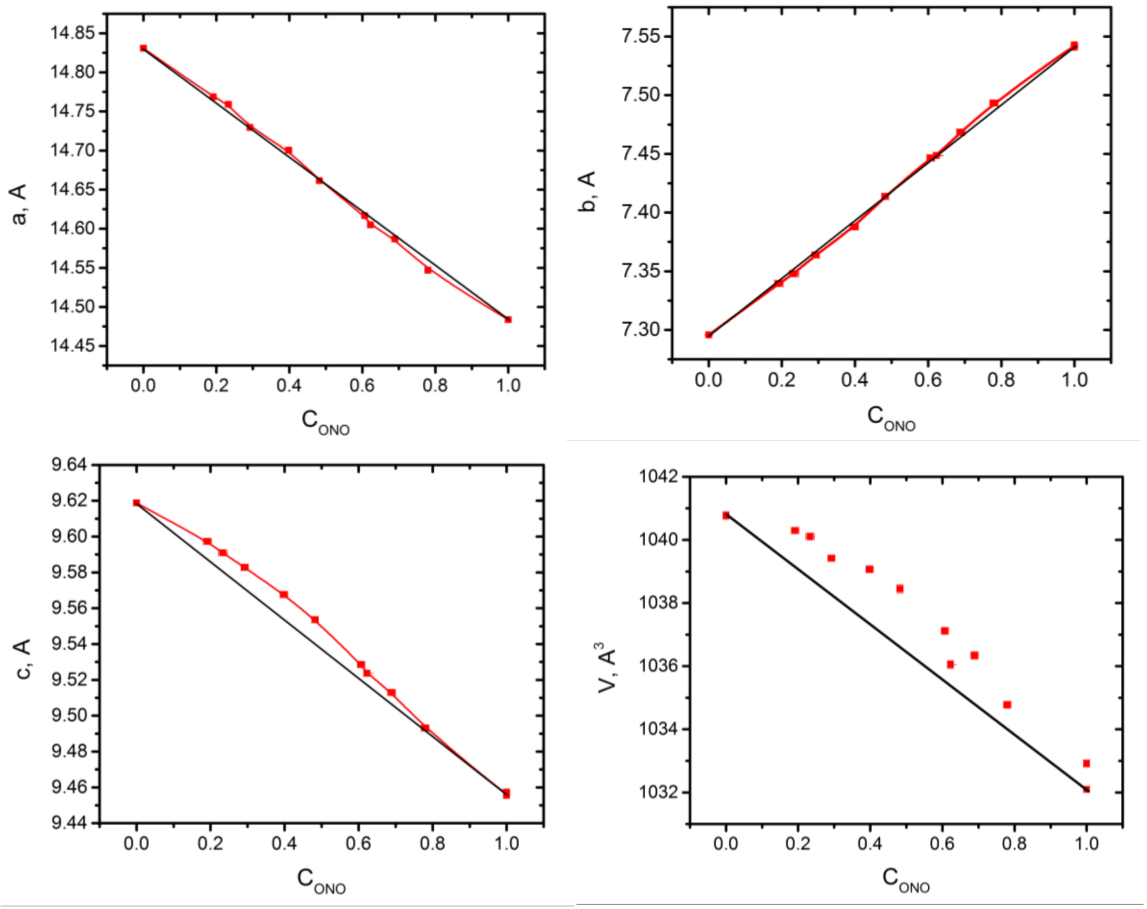

Figure S2. Dependencies of cell parameters *a*, *b*, *c* and cell volume *V* on the content of 1-*O* (nitrito) isomer in the crystals of $[Co(NH_3)_5NO_2]Cl(NO_3)$.

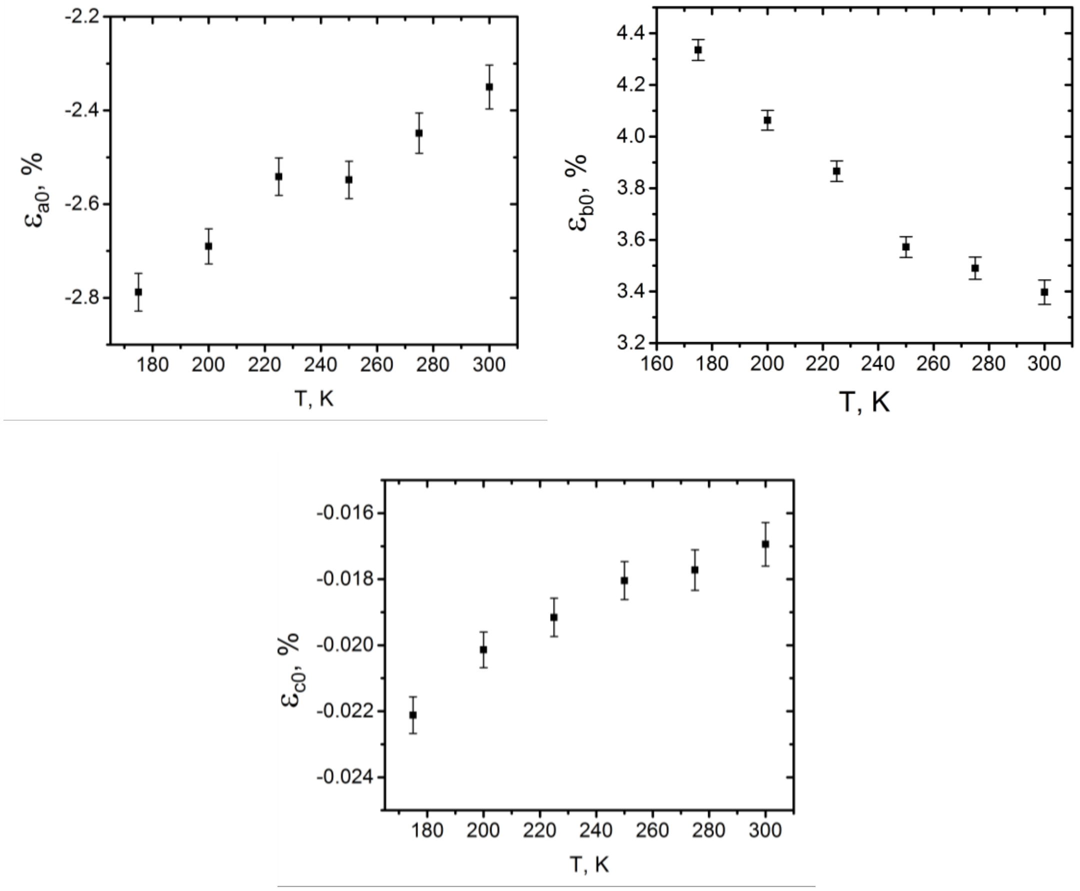

Figure S3. Temperature dependencies of the relative changes of the cell parameters *a* (a), *b* (b), *c* (c) caused by complete phototransformation from 1-*N* to 1-*O*.

a b

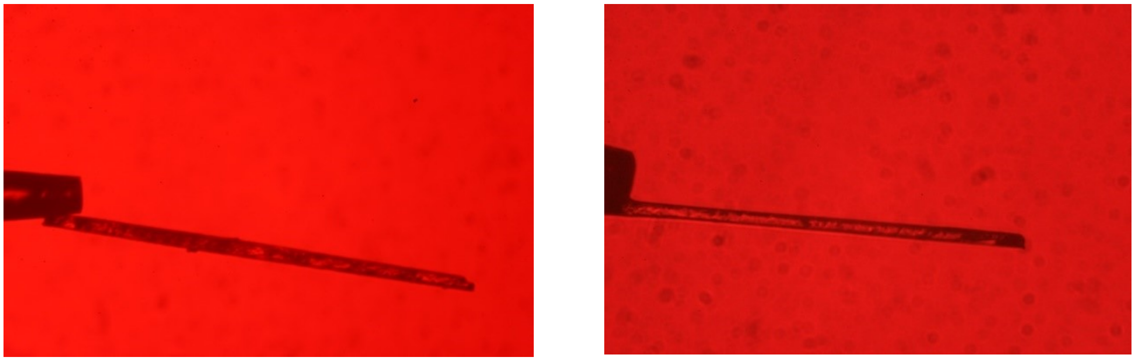

Figure S4. Crystals attached to the capillary and used in the bending experiments: crystal 1 (a), crystal 2 (b).

a

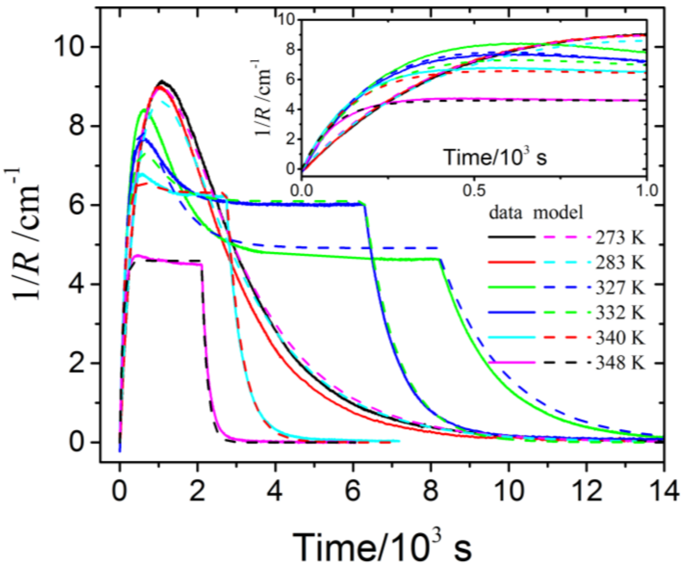

b

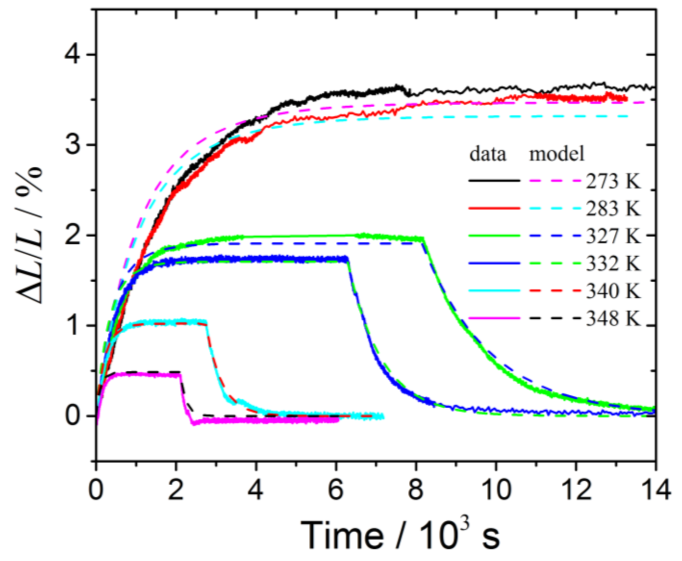

Figure S5. The kinetics of changing the curvature (a) and the length (b) of crystal 2 (for comparison with those for crystal 1 see Figures 6ab). Experimental data (solid lines) and quantitative description of the kinetics using the model with constant coefficients (dashed lines) described by eqs 4, 8 and 11

a

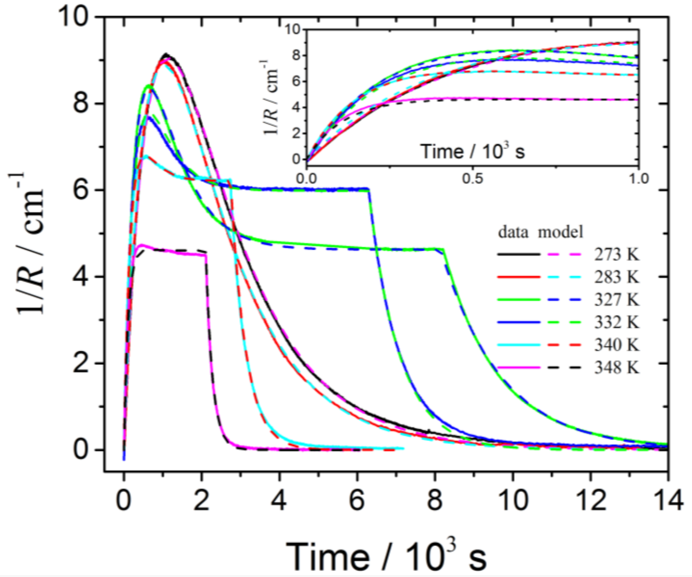

b

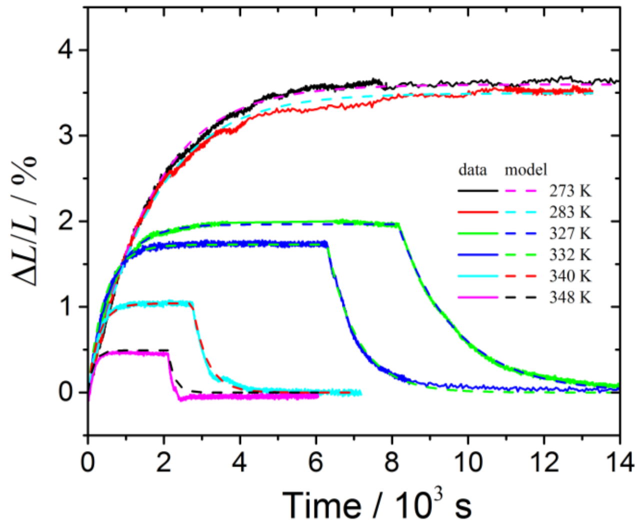

Figure S6. The kinetics of changing the curvature (a) and the length (b) of crystal 2 (solid lines) and quantitative description of the kinetics (dashed lines) using a model with the coefficients depending on the transformation degree described by eqs 15 and 16

Supporting tables

Table S1. Combination of crystal samples and temperatures used to verify the reproducibility of the measurements under conditions of uniform both-sides irradiation of single crystal of 1-*N* and thermal reversion of the resulting 1-*O*

Crystal length	Temperature / K					
	311	316	320	326	328	336
1700 µm	•			•		
1449 µm			•			
625 µm						••
836 µm			•		•	
650 µm		•				
446 µm		•				

Table S2. Data collection and refinement details for solid solutions $[Co(NH_3)_5NO_2]ClNO_3/[Co(NH_3)_5ONO]ClNO_3$[a]

No of experiment	1	2	3	4
ONO rate in solid solution with ESD	0.62273 (0.00852)	1	0.77971 (0.01073)	0.68906 (0.01077)
Crystal data				
a, *b*, *c* (Å)	14.6048 (15)<br>7.4486 (4)<br>9.5237 (5)	14.4710 (9)<br>7.5428 (3)<br>9.4556 (5)	14.5467 (12)<br>7.4933 (4)<br>9.4931 (5)	14.5868 (13)<br>7.4684 (3)<br>9.5130 (5)
V (Å^3)	1036.05 (13)	1032.10 (9)	1034.78 (11)	1036.34 (12)
No. of reflections for cell measurement	526	671	560	510
θ range (°) for cell measurement	2.0–16.2	2.1–16.2	2.0–16.5	2.0–16.2
μ (mm^{-1})	0.43	0.43	0.43	0.43
Data collection				
No. of measured, independent and observed [$I > 2\sigma(I)$] reflections	3398, 1050, 820	4527, 1111, 871	4495, 1122, 828	4452, 1120, 782
R_{int}	0.043	0.057	0.055	0.071
θ values (°)	θ_{max} = 14.9, θ_{min} = 2.0	θ_{max} = 14.9, θ_{min} = 2.0	θ_{max} = 14.9, θ_{min} = 2.0	θ_{max} = 14.9, θ_{min} = 2.0
$(\sin \theta/\lambda)_{max}$ (Å^{-1})	0.625	0.625	0.625	0.625
Range of *h*, *k*, *l*	h = -13→17, k = -9→9, l = -11→11	h = -18→15, k = -9→9, l = -11→11	h = -15→18, k = -9→9, l = -11→11	h = -16→18, k = -9→9, l = -11→11
Refinement				
$R[F^2 > 2\sigma(F^2)]$, $wR(F^2)$, S	0.042, 0.120, 1.19	0.049, 0.145, 1.20	0.056, 0.171, 1.13	0.059, 0.174, 1.14
No. of reflections	1050	1111	1122	1120
No. of parameters	95	76	95	95
No. of restraints	90	0	84	90
$\Delta\rho_{max}$, $\Delta\rho_{min}$ (e Å^{-3})	0.52, -0.51	0.70, -0.83	0.60, -1.06	1.24, -1.00

No of experiment	5	6	7	8
ONO rate in solid solution with ESD	0.60669 (0.01007)	0.39822 (0.01042)	0.29218 (0.01003)	0.23312 (0.0118)
Crystal data				
a, *b*, *c* (Å)	14.6166 (11)<br>7.4465 (3)<br>9.5286 (5)	14.7005 (12)<br>7.3877 (3)<br>9.5676 (5)	14.7295 (11)<br>7.3639 (3)<br>9.5828 (4)	14.7589 (12)<br>7.3480 (3)<br>9.5909 (4)

V (Å^3)	1037.12 (11)	1039.07 (11)	1039.42 (10)	1040.11 (11)
No. of reflections for cell measurement	588	640	705	631
θ range (°) for cell measurement	2.0–16.2	2.0–16.2	2.0–16.2	2.0–15.9
μ (mm^{-1})	0.43	0.43	0.43	0.43
Data collection				
No. of measured, independent and observed [$I > 2\sigma(I)$] reflections	4384, 1121, 811	4406, 1121, 813	4487, 1125, 841	4458, 1126, 810
R_{int}	0.082	0.065	0.055	0.067
θ values (°)	θ_{max} = 14.9, θ_{min} = 2.0	θ_{max} = 14.9, θ_{min} = 2.0	θ_{max} = 14.9, θ_{min} = 2.0	θ_{max} = 14.9, θ_{min} = 2.0
(sin $\theta/\lambda)_{max}$ (Å^{-1})	0.625	0.625	0.625	0.625
Range of h, k, l	h = -18→15, k = -9→9, l = -11→11	h = -16→18, k = -9→9, l = -11→11	h = -16→18, k = -9→9, l = -11→11	h = -16→18, k = -9→9, l = -11→11
Refinement				
$R[F^2 > 2\sigma(F^2)]$, $wR(F^2)$, S	0.056, 0.168, 1.13	0.051, 0.152, 1.07	0.050, 0.149, 1.11	0.057, 0.189, 1.13
No. of reflections	1121	1121	1125	1126
No. of parameters	95	95	95	95
No. of restraints	90	84	90	84
$\Delta\rho_{max}$, $\Delta\rho_{min}$ (e Å^{-3})	1.14, -0.76	0.89, -0.76	0.75, -0.89	0.89, -1.01

No of experiment	9	10	11	12
ONO rate in solid solution with ESD	0.19162 (0.01129)	0	1	0.48228 (0.00903)
Crystal data				
a, b, c (Å)	14.7688 (10)<br>7.3395 (3)<br>9.5973 (4)	14.8308 (13)<br>7.2957 (4)<br>9.6188 (5)	14.4836 (12)<br>7.5409 (4)<br>9.4574 (5)	14.6615 (14)<br>7.4138 (4)<br>9.5536 (5)
V (Å^3)	1040.30 (10)	1040.77 (12)	1032.92 (11)	1038.45 (13)
No. of reflections for cell measurement	618	645	625	629
θ range (°) for cell measurement	2.0–16.3	2.0–16.4	2.0–15.8	2.0–17.0
μ (mm^{-1})	0.43	0.43	0.43	0.43
Data collection				
No. of measured, independent and	4458, 1120, 804	4451, 1123, 825	4477, 1108, 826	4491, 1119, 832

observed [$I > 2\sigma(I)$] reflections				
R_{int}	0.067	0.069	0.064	0.058
θ values (°)	θ_{max} = 14.9, θ_{min} = 2.0	θ_{max} = 14.9, θ_{min} = 2.0	θ_{max} = 14.9, θ_{min} = 2.0	θ_{max} = 14.9, θ_{min} = 2.0
$(\sin \theta/\lambda)_{max}$ (Å^{-1})	0.625	0.625	0.625	0.625
Range of h, k, l	h = -16→18, k = -9→9, l = -11→11	h = -15→18, k = -9→9, l = -12→12	h = -15→17, k = -9→9, l = -11→11	h = -15→18, k = -9→9, l = -11→11
Refinement				
$R[F^2 > 2\sigma(F^2)]$, $wR(F^2)$, S	0.055, 0.186, 1.16	0.049, 0.150, 1.17	0.055, 0.175, 1.14	0.046, 0.132, 1.12
No. of reflections	1120	1123	1108	1119
No. of parameters	95	76	76	95
No. of restraints	84	0	0	90
$\Delta\rho_{max}$, $\Delta\rho_{min}$ (e Å^{-3})	0.71, -1.16	1.03, -0.89	0.60, -0.94	0.78, -0.71

[a]For all structures: chemical formula, $ClCoH_{15}N_7O_5$, M_r = 287.57, orthorhombic, *Pnma*, Z = 4, crystal size 0.25 × 0.05 × 0.01 mm, T = 295 K, λ = 0.4109 Å. The absorption was corrected for by multiscan methods. The hydrogen atom parameters were constrained.

Table S3. Variable-temperature cell refinement for $[Co(NH_3)_5ONO]ClNO_3$ and $[Co(NH_3)_5NO_2]ClNO_3$

	$[Co(NH_3)_5ONO]ClNO_3$ (*Pnma*)					
	300 K	275 K	250 K	225 K	200K	175 K
Cell parameters *a, b, c*, Å	14.4851 (56)<br>7.5269 (28)<br>9.4237 (50)	14.4456 (48)<br>7.5226 (24)<br>9.4218 (44)	14.4136 (44)<br>7.5121 (22)<br>9.4217 (41)	14.3924 (44)<br>7.5119 (22)<br>9.4165 (41)	14.3612 (41)<br>7.5066 (21)<br>9.4100 (38)	14.3275 (39)<br>7.5013 (20)<br>9.3995 (34)
Cell volume, $Å^3$	1027.44 (78)	1023.85 (68)	1020.14 (62)	1018.05 (62)	1014.43 (57)	1010.21 (53)
2θ range	5.17→46.11	5.21→46.26	5.14→46.30	5.14→46.60	5.14→46.49	5.21→47.41
No of reflections for cell refinement	468	530	560	592	614	632
	$[Co(NH_3)_5NO_2]ClNO_3$ (*Pnma*)					
	300 K	275 K	250 K	225 K	200K	175 K
Cell parameters *a, b, c*, Å	14.8337 (41)<br>7.2796 (20)<br>9.5861 (39)	14.8082 (42)<br>7.2689 (20)<br>9.5918 (39)	14.7905 (39)<br>7.2530 (19)<br>9.5948 (37)	14.7677 (40)<br>7.2323 (19)<br>9.6004 (38)	14.7582 (37)<br>7.2135 (18)<br>9.6034 (36)	14.7384 (44)<br>7.1896 (21)<br>9.6121 (41)
Cell volume, $Å^3$	1035.14 (58)	1032.45 (58)	1029.28 (55)	1025.38 (56)	1022.36 (52)	1018.53 (61)
2θ range	4.95→47.65	5.01→47.78	5.09→47.84	5.05→47.88	5.06→47.69	5.05→47.70
No of reflections for cell refinement	723	748	771	781	785	771

Table S4. Dependence of the thermal isomerization rate constant, k_{th}, on temperature

Temperature/ K	k_{th}/s^{-1}
by the uniform deformation method	
311	1.0(6)×10^{-4}
316	1.(7)×10^{-4}
316	1.(4)×10^{-4}
320	3.(2)×10^{-4}
320	3.1(4)×10^{-4}
326	5.(7)×10^{-4}
328	7.(0)×10^{-4}
336	1.5(8)×10^{-3}
336	1.7(3)×10^{-3}
by the unbending method	
299	2.0(7)×10-5
326	6.5(4)× 10^{-4}
327	6.8(9)× 10^{-4}
332	1.2(8)×10^{-3}
333	1.4(7)×10^{-3}
340	2.5(6)×10^{-3}
348	5.7(3)×10^{-3}
351	7.8(7)×10^{-3}
357	1.3(7)×10^{-2}
